# Mature Dendritic Cell-Derived Extracellular Vesicles are Potent Mucosal Adjuvants for Influenza Hemagglutinin Vaccines

**DOI:** 10.1021/acsnano.5c08831

**Published:** 2025-07-01

**Authors:** Chunhong Dong, Lai Wei, Wandi Zhu, Joo Kyung Kim, Ye Wang, Priscilla Omotara, Arini Arsana, Bao-Zhong Wang

**Affiliations:** Center for Inflammation, Immunity & Infection, 439338 Georgia State University Institute for Biomedical Sciences, Atlanta, Georgia 30302, United States

**Keywords:** dendritic cell-derived extracellular vesicles, recombinant protein vaccines, intranasal vaccination, immunoenhancing effect, influenza cross-protection

## Abstract

Immune cell-derived extracellular vesicles (EVs) possess intrinsic immunomodulatory properties, making them potential vaccine adjuvants. Here, we show that EVs from mature bone marrow-derived dendritic cells (mDC-EVs), rather than those from immature dendritic cells (imDC-EVs), are potent mucosal adjuvants for influenza hemagglutinin (HA) vaccines. In vitro, mDC-EVs exhibited intriguing immune-stimulating effects on various antigen-presenting cells, including DCs, macrophages, and B cells. Furthermore, intranasal immunization with mDC-EVs-adjuvanted A/Aichi/2/1968 (H3N2) HA (H3+mDC-EVs) significantly enhanced and expanded both systemic and mucosal antibody and cellular immune responses in female Balb/c mice. These responses offered complete protection against bodyweight loss following homologous and heterologous virus challenges. Mechanistically, H3+mDC-EVs immunization promoted enhanced airway immune cell recruitment, distinct antigen cellular uptake, and rapid activation of B and T cells within 24 h. It also induced robust germinal center reactions and antigen-experienced memory T-cell responses in lung-draining mediastinal lymph nodes 14 days postimmunization. Given their biocompatibility and solid adjuvanticity, mDC-EVs represent a promising adjuvant candidate for mucosal vaccine development.

## Graphical abstract

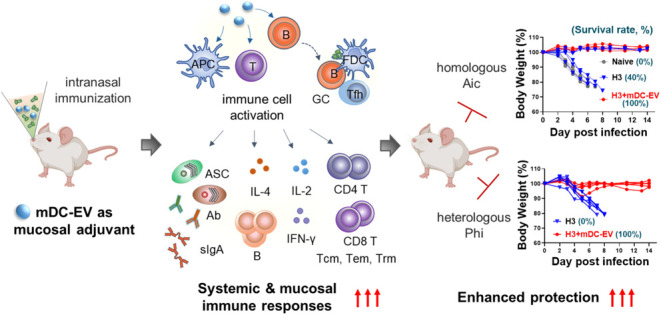

Given the limited effectiveness of existing seasonal influenza vaccines against mismatched, evolved strains,^1^ next-generation cross-protective influenza vaccines are urgently needed. Recombinant protein vaccines have attracted intensive attention in vaccine development due to their safety, ease of large-scale manufacturing, and affordability.^2^ Compared with virus-based vaccines, protein subunit vaccines are safer and can be designed to target specific pathogen components, leading to more focused immune responses. Protein vaccines also have better stability than mRNA vaccines which require ultracold storage. Mucosal immunization represents a promising strategy against respiratory infectious diseases by inducing mucosal immunity that is essential for preventing the infection and transmission of respiratory pathogens, while also exhibiting potential cross-protection.^3^ However, the effectiveness of protein vaccines administered mucosally is limited by the mucosal immune tolerance environment and their inherent weak ability of immune activation, highlighting the need for safe and effective mucosal adjuvants.

Extracellular vesicles (EVs) are cell-derived, lipid-bound vesicular particles that exhibit inherent safety and biocompatibility and play crucial roles in intercellular communication and modulating biological responses.^4,5^ Increasing studies suggest that EVs can modulate immune responses, which can be either stimulatory or suppressive depending on their respective cellular origins and conditions.^6,7^ Notably, EVs derived from dendritic cells (DCs)—the most potent professional antigen-presenting cells—have garnered significant attention in immunotherapy and vaccine development due to harboring a variety of immunologically active molecules crucial for effective antigen presentation, as well as cell adhesion and fusion.^8^ DC-EVs carrying tumor peptide/MHC complexes or loaded with tumor antigens have demonstrated the capability to induce cytotoxic T lymphocytes and eradicate or suppress the growth of murine tumors *in vivo*, representing a promising alternative to adoptive cell therapy.^9–12^ Later, EVs isolated from LPS-stimulated human THP-1 cells were shown to enhance IFN-γ-mediated cellular immune responses to subcutaneously administered hepatitis B antigens.^13^ More recently, extracellular blebs derived from genetically engineered spike protein-expressing DC2.4 cells elicited potent neutralizing antibodies against pseudotyped SARS-CoV-2.^14^ Despite these studies reporting the adjuvanticity of DC-EVs for conventional systemically administered vaccines, their potential in mucosal vaccines targeting respiratory infectious diseases and the underlying mechanisms of action remain unexplored.

This study investigated the potential of DC-EVs as mucosal adjuvants for influenza hemagglutinin (HA) vaccines. We isolated EVs from mature (mDC-EVs) and immature (imDC-EVs) bone marrow-derived DCs, characterized their immunostimulatory properties in vitro, and evaluated their adjuvanticity in vivo. Intranasal administration of female Balb/c mice with recombinant A/Aichi/2/1968­(H3N2) HA (H3) protein combined with mDC-EVs (H3+mDC-EVs) demonstrated enhanced airway immune cell recruitment, distinct antigen cellular uptake, and early T and B cell activation compared to H3 alone. Moreover, H3+mDC-EVs immunization triggered significantly stronger germinal center reactions (GC B and Tfh) and generated robust central and effector memory T cell (Tcm and Tem) responses in lung-draining mediastinal lymph nodes (mLNs). A two-dose immunization regimen with H3+mDC-EVs induced multifaceted and broadly reactive humoral and cellular immune responses against divergent influenza viruses or HA subtypes, conferring complete protection against homologous and heterologous influenza challenges. These findings highlight the promise of mDC-EVs as an attractive adjuvant or target for mucosal influenza vaccination.

## Results

### Isolation and In Vitro Characterization of EVs Derived from BMDCs

EVs derived from immature or mature bone marrow-derived DCs (BMDCs) were isolated using differential ultracentrifugation. The resulting DC-EVs exhibited a relatively uniform particle distribution, with Number-mean sizes of 120 ∼ 130 nm, *Z*-average sizes of 150 ∼ 170 nm, PDI indexes below 0.2, and surface charges of −31 to −36 mV (Figures 1A,B and S1A,B). Scanning electron microscopy (SEM) was used to characterize the morphology of mDC-EVs. The mDC-EVs are well-dispersed, spherical nanoparticles (Figure S1C). The Western blot assay verified the presence of EV marker proteins, including CD81, TSG101, and Alix, on mDC-EVs and imDC-EVs (Figures 1C and S1D). Considering that LPS was employed as a stimulus to promote DC maturation, we further evaluated its influence on final purified EVs using the Pierce chromogenic endotoxin quant kit (Figure S1E). No significant difference was observed between mDC-EVs and imDC-EVs groups, indicating that the LPS treatment during cell culture did not have a substantial effect on the final EV products.

**Figure 1:**
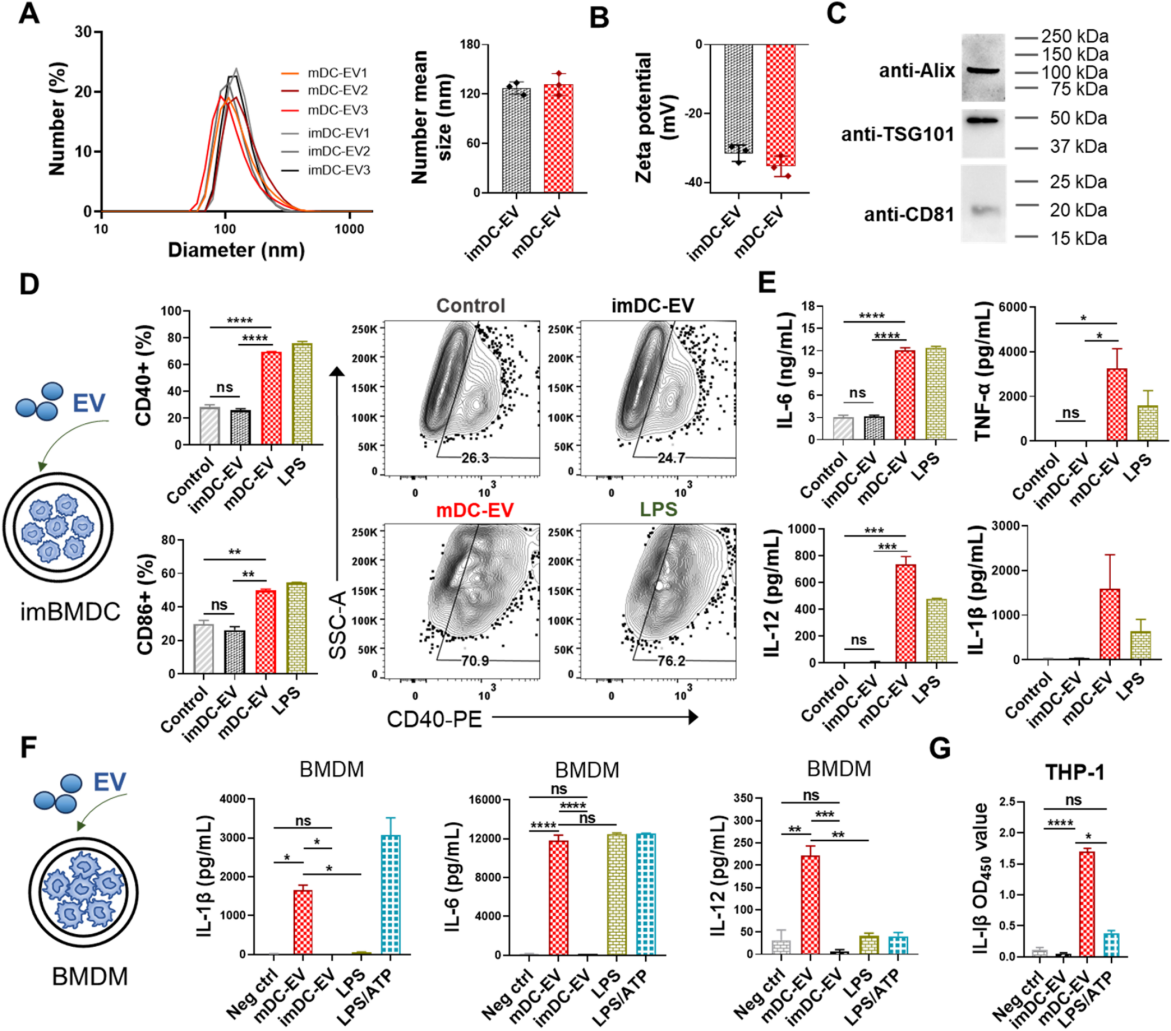
Preparation and in vitro characterization of DC-EVs. (A, B) Size distribution and zeta potential of the obtained DC-EV particles determined by dynamic light scattering. (C) Western blotting analysis of the EV surface markers on mDC-EV particles. (D) The immunostimulant effects of EVs on immature BMDC. The expression of CD40 and CD86 maturation markers on BMDC was characterized by flow cytometry under the indicated stimulations. (E) The secreted cytokine (IL-6, IL-12, TNF-α, IL-1β) levels in BMDC cell cultures. (F) The immunostimulant effects of EVs on BMDM cells. The IL-1β, IL-6, and IL-12 levels in BMDM cell cultures. (G) The human IL-1β levels in THP-1 macrophage cell cultures under the indicated stimulations. Data are presented as mean ± SEM (*n* = 2 samples for D-G). Statistical significance was analyzed by one-way ANOVA followed by Tukey’s multiple comparison tests (*p* > 0.05, ns, not significant; *p < 0.05; **p < 0.01; ***p < 0.001; ****p < 0.0001).

A well-established characteristic of numerous vaccine adjuvants is their ability to induce DC maturation and stimulate the secretion of inflammatory cytokines. Here, we investigated the immunostimulatory effects of purified DC-EVs on murine BMDCs and JAWSII cells. The results showed that mDC-EVs induced BMDC maturation, as evidenced by significantly increased expression of DC maturation markers CD40 and CD86 (Figures 1D and S2A,B), along with the secretion of inflammatory cytokines IL-6, IL-12, TNF-α, and IL-1β (Figure 1E). Similarly, the addition of mDC-EVs enhanced CD86 expression on JAWSII cells, whereas H3 alone did not (Figure S2C,D). By contrast, imDC-EVs did not exhibit such effects.

Inflammasomes are also crucial players in innate immunity and can shape adaptive immune responses to vaccination and pathogen infection.^15,16^ To investigate whether mDC-EVs can induce inflammasome activation, we cocultured bone marrow-derived macrophages (BMDMs) and macrophages derived from human THP-1 cells with purified DC-EVs. We assessed the downstream secretion of IL-1β following established protocols.^15,17^ In parallel, we also measured the levels of secreted IL-6 and IL-12 in the BMDM cultures. Our results demonstrated that sole LPS stimulation could induce IL-6 and IL-12, but not apparent IL-1β secretions in BMDMs (Figures 1F and S2E), while LPS stimulation followed by subsequent ATP treatment (LPS/ATP, the positive control group) effectively activated inflammasomes. mDC-EVs, rather than imDC-EVs, induced significant IL-1β secretion in BMDM cultures, indicating activation of inflammasomes, and enhanced IL-6 and IL-12 secretion. Similarly, robust IL-1β secretion was observed from mDC-EVs- but not imDC-EVs-treated THP-1-derived macrophages (Figure 1G). These results suggest that mDC-EVs, but not imDC-EVs, are potent stimulators for inflammation activation. To further investigate the possible triggers of the inflammasome activation, we used inhibitors in the stimulation process of THP-1 macrophages (Figure S2F). We found that the addition of cathepsin B inhibitor CA-074-Me or K+ channel inhibitor amiodarone did not impede mDC-EV-induced IL-1β secretion, which rules out the involvement of endosomal disruption-associated cathepsin B release or K+ efflux. Further analyses were needed to elucidate the specific activation pathways.

### Systemic Antibody Cross-Reactivity and Antibody-Secreting Cell Responses

Acknowledging the immunostimulatory effects of mDC-EVs, we proceeded to investigate their potential as a mucosal adjuvant to boost vaccine immunogenicity. Female Balb/c mice were intranasally immunized with H3+mDC-EVs or soluble H3 alone by a two-dose regimen (Figure 2A). No mice displayed any clinical symptoms, such as body weight loss post-vaccination, indicating a favorable safety profile (Figure S3A). We analyzed H3-specific IgG levels in prime sera 3 and 10 weeks post-prime vaccination and found that H3 alone is weakly immunogenic when administered intranasally, as evidenced by the low levels of H3-specific serum IgG (Figure S3B). By contrast, H3+mDC-EVs induced significant antigen-specific IgG antibodies, revealing the mDC-EV’s robust adjuvant effects in vivo. Moreover, significantly higher IgG levels were observed in 10- versus 3-week immune sera, indicating durable antibody production following priming. Boost sera were collected 3 weeks post-boost immunization. Similarly, significantly boosted H3-specific IgG antibodies were detected in the H3+mDC-EVs group (Figure 2A). Antibody subtype analysis suggested that H3-specific IgG1, IgG2a, and IgG2b antibodies were all increased in the H3+mDC-EVs group (Figure 2B). However, no significant differences were observed in the H3-specific IgG1/IgG2a ratios between the H3 and H3+mDC-EVs groups (Figure S3C). Robust serum IgG was detected against the homologous Aic virus (Figure 2C).

**Figure 2:**
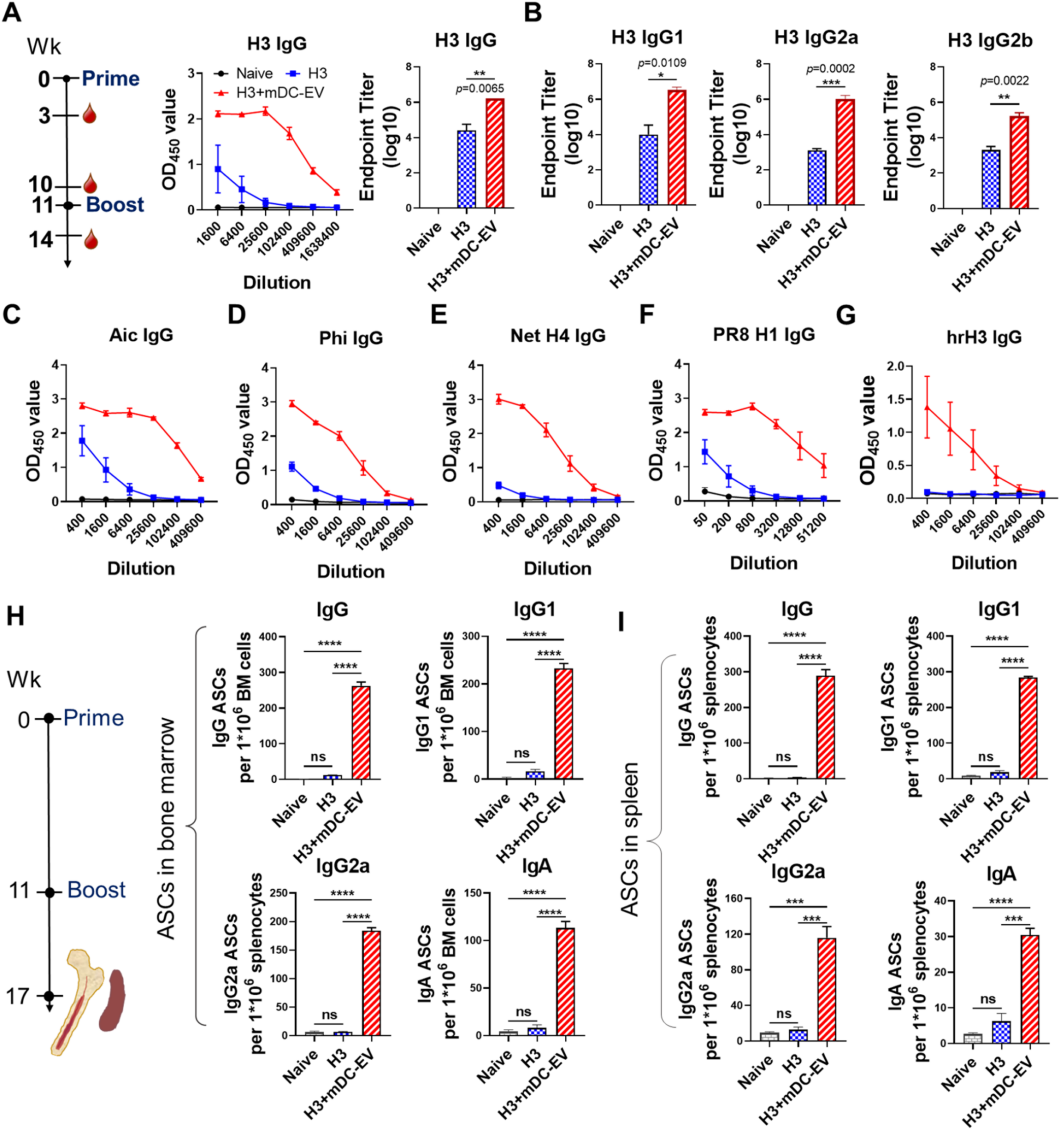
Antibody and antibody-secreting cell (ASC) responses. (A, B) H3-specific IgG, IgG1, IgG2a, and IgG2b antibody levels in boost immune sera. (C–G) IgG cross-reactivity against Aic, Phi, Net H4, hrH3, and PR8 H1 in boost immune sera. (H, I) Antigen-specific IgG, IgG1, IgG2a, and IgA ASC frequencies in mouse bone marrows and spleens. 4 μg/mL of recombinant H3 proteins were used to coat the ELISpot plates. Spleens and bone marrow were collected 6 weeks post-boosting immunization. Data are presented as mean ± SEM (*n* = 3 mice per group). Statistical significance was analyzed by one-way ANOVA followed by Tukey’s multiple comparison tests (*p* > 0.05, ns, not significant; *p < 0.05; **p < 0.01; ***p < 0.001; ****p < 0.0001).

Antibody cross-reactivity is crucial in providing broad protection against mutated influenza viruses. We evaluated the boost immune serum antibody cross-reactivity using heterologous, cross-subtype, and cross-group virus strains or HAs. Our results showed that IgG antibodies induced by H3+mDC-EVs immunization exhibited significantly enhanced cross-reactivity against heterologous Phi (H3N2) and Wis (H3N2), and heterosubtypic Net H4 and Anh H7, compared to the H3 immunization group (Figures 2D,2E and S3D–F). We analyzed the antibody subtype profiles and observed that H3+mDC-EVs immunization boosted both IgG1 and IgG2a antibodies against Phi and Anh H7 (Figure S3D,F). Moreover, H3+mDC-EVs immunization also generated significant antibodies against HAs of cross-group strains, including PR8 H1, Net H9, and Swe H12 from group 1 (Figures 2F and S3G,H). The stalk domain of influenza HA is an attractive immune target for developing universal influenza vaccines due to its conserved nature across HA subtypes, in contrast to the highly variable HA head.^18,19^ Antibodies targeting the HA stalk domain are highly effective in inducing Fc-mediated antibody effector functions, including antibody-dependent cell-mediated cytotoxicity (ADCC).^20^ We detected antibody levels against head-removed Aic HA (hrH3). Immunization with H3+mDC-EVs promoted the generation of hrHA3-specific IgG, IgG1, and IgG2a antibody levels in boost immune sera (Figures 2G and S3I). Thus, the mDC-EVs are intriguing adjuvants that can enhance antibody magnitude, diversity, and cross-reactivity. However, subsequent microneutralization assays revealed no detectable cross-neutralization activity against Phi or rSH viruses, consistent with our previous findings.^21,22^

Antibodies are secreted by antibody-secreting cells (ASCs), which can be either short-lived plasma cells and plasmablasts or long-lived plasma cells (LLPCs). The bone marrow (BM) is vital in maintaining LLPCs that produce sustained antibody responses.^23^ We assessed the ASC frequencies in bone marrow by the B-cell ELISpot assay 6 weeks post-boosting immunization, and found that vaccination with H3 alone induced poor antigen-specific ASC populations in the bone marrow, with no significant difference observed versus the naïve group (Figure 2H). In contrast, immunization with H3+mDC-EVs induced significant IgG, IgG1, IgG2a, and IgA ASCs in the bone marrow, aligning with the robust antibody responses observed in immune sera. Similar results were observed for splenic ASCs (Figure 2I). The H3+mDC-EVs group generated significantly more IgG, IgG1, IgG2a, and IgA ASCs in the spleens than the H3 alone and naive groups.

### Cross-Reactive Systemic Cellular Immune Responses

Cellular immunity is critical in restricting viral replication and eliminating virus-infected cells by producing cytokines and effector molecules.^24^ T-cell cross-reactivity is crucial in combating rapidly evolving pathogens, such as influenza. Cross-reactive T cells are essential correlates of cross-protection.^25^ Here, we evaluated T cell cross-reactivity by determining the cytokine-secreting cells in the spleens using a T cell ELISpot assay with different HAs as stimuli 6 weeks post-boosting immunization (Figure 3). Our results revealed that H3 immunization alone failed to elicit antigen-specific cytokine-secreting cells in the mouse spleens. In contrast, immunization with H3+mDC-EVs induced robust H3-specific IL-2-, IL-4-, and IFN-γ-secreting T cell responses (Figure 3A,E,I). Moreover, the induced T cells demonstrated significant cross-reactivity to heterosubtypic Net H4 (Figure 3B,F,J), Anh H7 (Figure 3C,G,K), and were also responsive to the conserved hrH3 (Figure 3D,H,L). The robust IL4- and IFN-γ-secreting cell responses were consistent with the observed enhancement of both Th2-type IgG1 and Th1-type IgG2a antibodies (Figure 2). These findings suggested the high potency of mDC-EVs in enhancing cellular responses.

**Figure 3:**
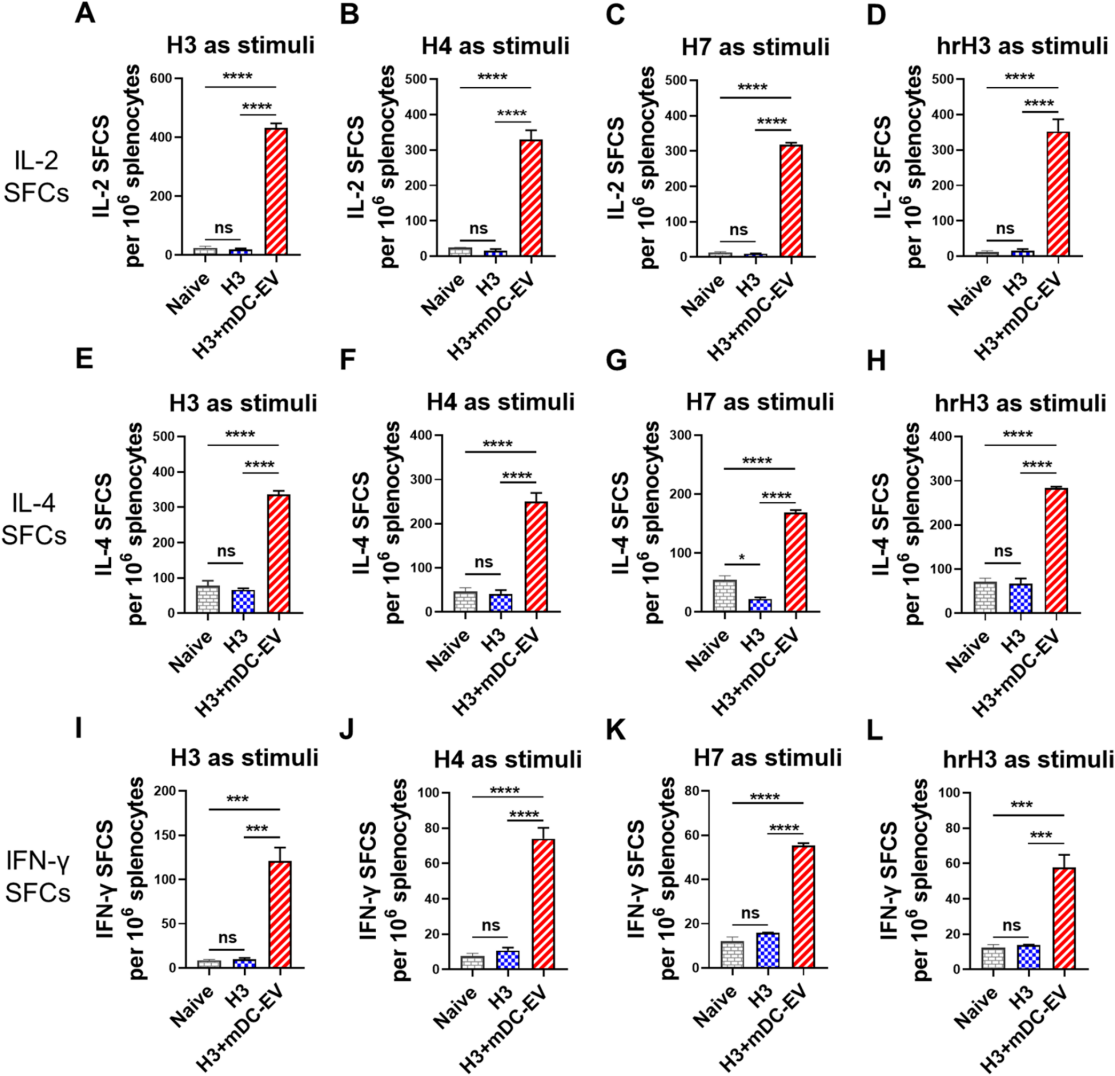
Cross-reactive cellular immune responses. (A–D) IL-2-secreting splenocytes. (E–H) IL-4-secreting splenocytes. (I–L) IFN-γ-secreting splenocytes. Spleens were collected 6 weeks post-boosting immunization. Four μg/mL of recombinant H3, H4, H7, or hrH3 proteins were used as the stimuli. Data are presented as mean ± SEM (*n* = 3 mice per group). Statistical significance was analyzed by one-way ANOVA followed by Tukey’s multiple comparison tests (*p* > 0.05, ns, not significant; *p < 0.05; **p < 0.01; ***p < 0.001; ****p < 0.0001).

### Mucosal Antibody and Cellular Immune Responses

An effective mucosal vaccine should induce mucosal immunity in the respiratory tract, the first line of defense against respiratory infectious diseases. Secretory IgA (sIgA) at mucosal surfaces is essential for mucosal antibody immunity against respiratory viruses. We collected mouse nasal washes and bronchoalveolar lavage fluid (BALF) 6 weeks post-boosting immunization and determined sIgA levels. Immunization with H3+mDC-EVs significantly boosted H3-specific sIgA (Figure 4A,4E), aligning with the elevated H3-specific IgA levels in immune sera (Figure S4A). Moreover, cross-reactive sIgA antibodies were detected against heterologous Phi, Wis or Wis HA, HK HA, heterosubtypic Net H4, Swe H10, and cross-group PR8 H1 and Swe H12 in both nasal washes (Figures 4B–4D and S4B) and BALF (Figures 4F–4H and S4C). We also determined antigen-specific IgG levels in BALF, the most common immunoglobulin in the lower respiratory tract. Our results suggest that immunization with H3+mDC-EVs elicited intense cross-reactive IgG levels against all detected HAs or inactivated viruses, including H3, Aic, Phi, Wis, Net H4, Anh H7, Swe H10, the conserved hrH3, and PR8 H1 and Swe H12 (Figure S5). These IgG and sIgA antibodies at mucosal sites can potentially prevent influenza viruses from spreading to the lower respiratory tract and deeper tissues.

**Figure 4:**
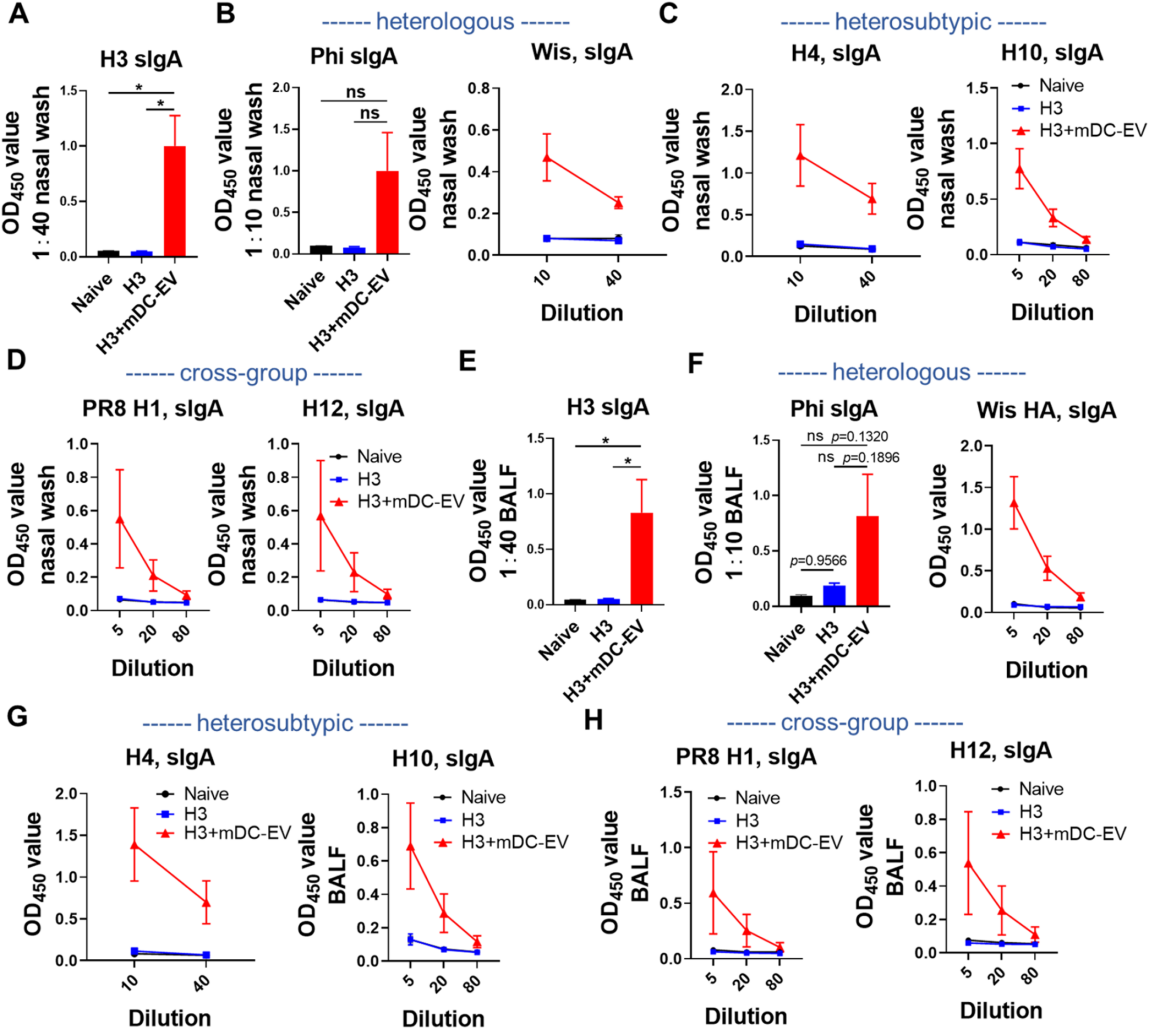
Mucosal antibody responses. (A–D) sIgA antibody responses in nasal washes against H3, heterologous Phi and Wis, heterosubtypic Net H4 and Swe H10, and cross-group PR8 H1 and Swe H12, respectively. (E–H) sIgA antibody responses in BALF against H3, Phi, Wis HA, Net H4, Swe H10, PR8 H1, and Swe H12, respectively. Nasal washes and BALF were collected 6 weeks post-boosting immunization. Data are presented as mean ± SEM (*n* = 3 mice per group). Statistical significance was analyzed by one-way ANOVA followed by Tukey’s multiple comparison tests (*p* > 0.05, ns, not significant; *p < 0.05; **p < 0.01; ***p < 0.001; ****p < 0.0001).

Local tissue-resident memory T cells (T_RM_) are a subset of memory T cells that reside in peripheral tissues, providing localized immunity and immunosurveillance. They play crucial roles in early and immediate defenses against viral infections.^4^ We collected cells from BALF and lung tissues of the immunized mice 6 weeks post-boosting immunization to evaluate local mucosal T-cell responses. Antigen-experienced CD44+ T cells were gated from BALF cells by flow cytometry (Figure S6A). Immunization with H3+mDC-EVs elicited elevated CD4+ and CD4+CD44+ T cell frequencies among the live singlet lymphocytes compared to the naïve and H3 groups, despite no significant difference observed in the frequencies of CD44+ populations inside CD4+ T cells (Figure 5A). We further evaluated airway T_RM_ cells using tissue-resident cell surface markers, CD69 and CD49a. CD69, a critical antagonist of S1PR1 (CD363) limiting the egress of cells into the bloodstream, is constitutively expressed on most lung T_RM_ (50–70%) under steady-state conditions.^26^ CD49a (integrin α1), another critical marker expressed in both CD4+ and CD8+ T_RM_ subsets, together with CD29 (integrin β1), forms the heterodimeric VLA-1, which can bind local collagen to maintain residency in the airways.^27^ CD49a plays a crucial role in T_RM_ tissue maintenance and retention.^28,29^ Our result showed that the H3+mDC-EVs group displayed double-positive CD69+CD49a+ CD4 and CD8 T cell populations, whereas the naïve and H3-immunization groups did not (Figure 5B,C). Additionally, a slight increase in the antigen-experienced CD4+CD44+ and CD8+CD44+ T cell populations was observed in the lungs of immunized compared to naïve mice (Figures 5D,5E and S6B).

**Figure 5:**
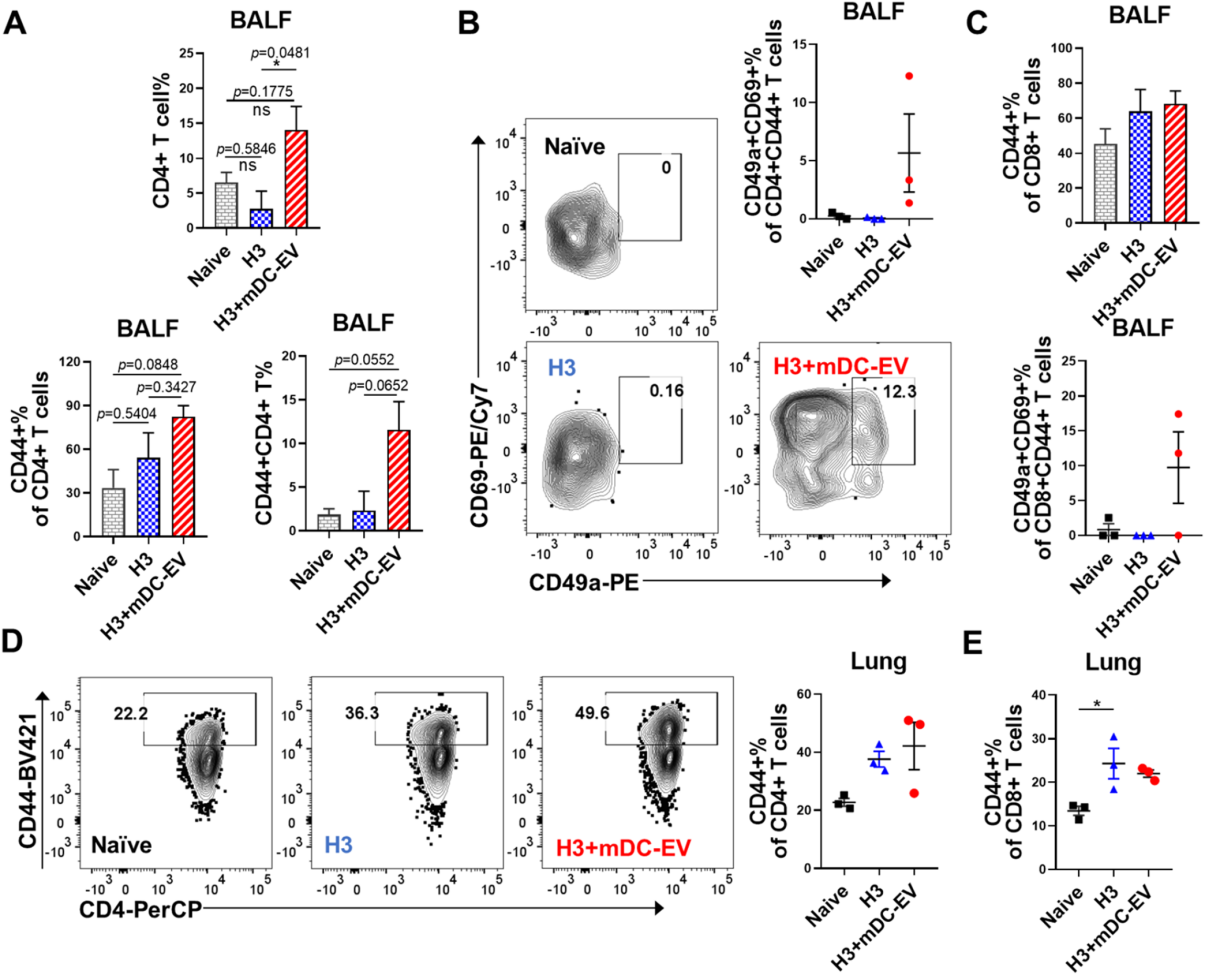
T cell immune responses in mouse airways and pulmonary tissues. (A) Comparison of CD4+ and CD4+CD44+ T cell populations in BALF. (B) Comparison of tissue-resident CD4+CD44+CD69+CD49a+ T_RM_ populations in BALF. (C) Comparison of CD8+CD44+ and tissue-resident CD8+CD44+CD69+CD49a+ T_RM_ populations in BALF. (D, E) Comparison of CD4+CD44+ and CD8+CD44+ T cell populations in mouse lungs. BALF and lung tissues were collected 6 weeks post-boosting immunization. Data are presented as mean ± SEM (*n* = 3 mice per group). Statistical significance was analyzed using one-way ANOVA followed by Tukey’s multiple comparison tests (*p* > 0.05, ns, not significant; *p < 0.05; **p < 0.01; ***p < 0.001; ****p < 0.0001).

### Protective Efficacy Against Homologous and Heterologous Influenza Virus Challenges

We challenged the immunized mice 4 weeks post-boosting immunization to evaluate the protective efficacy of H3+mDC-EVs versus H3. Mouse-adapted Aic virus (5 × LD_50_) was employed to assess the homologous protection efficacy. Upon Aic infection, naive mice lost body weight rapidly and succumbed within 7 days (Figure 6A,B). Immunization with H3 alone conferred partial protection, with a 40% survival rate; three out of five mice reached the humane endpoint within 8 days, and the other two survived. The significant variation in homologous protection efficiency observed within the H3-immunized mice was consistent with our previous findings.^21,22^ In contrast, all H3+mDC-EVs-immunized mice survived the challenge without apparent body weight loss. The H3+mDC-EVs group displayed significantly higher 7- and 14-day body weight area under the curve (AUC) than the H3 group post-Aic challenge (Figures 6C,D and S7A,B). Moreover, significant differences were observed in the survival rates among these three groups (Figure 6E). These results demonstrated that H3+mDC-EVs displayed superior homologous protection compared to H3 alone. We further compared the H3+imDC-EVs versus H3 groups. No significant differences were observed in H3-specific serum antibodies or protection efficacies against 5 × LD_50_ Aic challenges (Figure S7C–I). These results align with those observed from in vitro immunostimulating studies. Thus, mDC-EVs, rather than imDC-EVs, are potential mucosal adjuvants.

**Figure 6:**
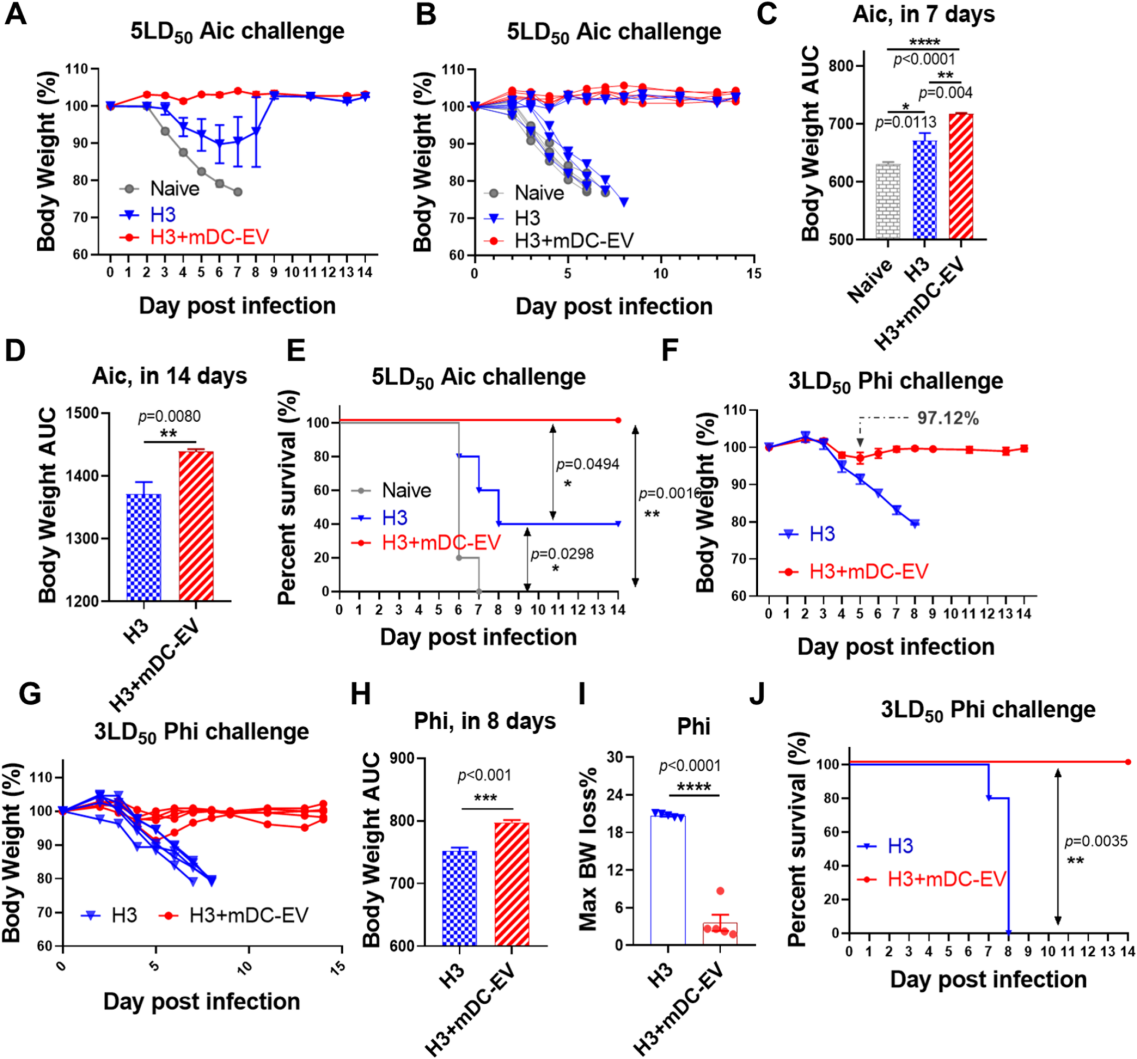
Protection efficacy post-virus challenges. (A, B) Mouse body weight curves post 5 × LD_50_ Aic challenge. (C, D) Mouse body weight AUC in 7 and 17 days post-Aic challenge. (E) Mouse survival rates post-Aic challenge. (F, G) Mouse body weight curves after the 3 × LD_50_ Phi challenge. (H) Mouse body weight AUC in 8 days post-Phi challenge. (I) The maximum body weight (BW) loss post-Phi challenge. (J) Mouse survival rates post-Phi infection. Data are presented as mean ± SEM (*n* = 5 mice per group). Statistical significance was analyzed by one-way ANOVA followed by Tukey’s multiple comparison tests (C), Student’s *t* test (D, H, and I), or Log-rank (Mantel-Cox) test (E, J) (*p* > 0.05, ns, not significant; *p < 0.05; **p < 0.01; ***p < 0.001; ****p < 0.0001).

To evaluate the efficacy of heterologous protection, we employed a 3 × LD_50_ dose of the mouse-adapted Phi (H3N2) for the challenge. In the H3 group, all mice succumbed to the challenge within 8 days. In contrast, the H3+mDC-EVs group demonstrated a 100% survival rate and near-complete protection against bodyweight loss (Figure 6E,F). Only one of the five mice in the H3+mDC-EVs group experienced noticeable body weight loss, but it recovered quickly. Besides, the H3+mDC-EVs group mice displayed a significantly higher 8-day body weight area AUC and lower maximum body weight loss (Max BW loss) than the H3 alone group post-Phi challenge (Figure 6H,I). The survival rate post-Phi challenge was also significantly higher in the H3+mDC-EVs group compared to the H3 group (Figure 6J).

Therefore, intranasal administration of HA alone proved insufficient to provide adequate protection against either homologous or heterologous influenza virus infection. In contrast, co-administration of mDC-EVs with H3 intranasally conferred robust protection, highlighting the potent mucosal adjuvant effects of mDC-EVs.

### Induction of Early Immune Responses

To gain insight into the mechanisms underlying the *in vivo* function of mDC-EVs, we assessed immune cell recruitment, antigen delivery and uptake profiles, and the initiation of early immune response on day 1 post-immunization. Female Balb/c mice were intranasally immunized with fluorescently labeled H3_AF700_ with or without mDC-EVs. Our previous study indicated that intranasally administered vaccines were mainly delivered to the lung tissues.^30^ Here, we found that abundant H3_AF700_ was delivered to the lung tissues in both H3_AF700_ and H3_AF700_+mDC-EVs immunized groups, and no significant differences were observed between these two groups (Figure 7A).

**Figure 7:**
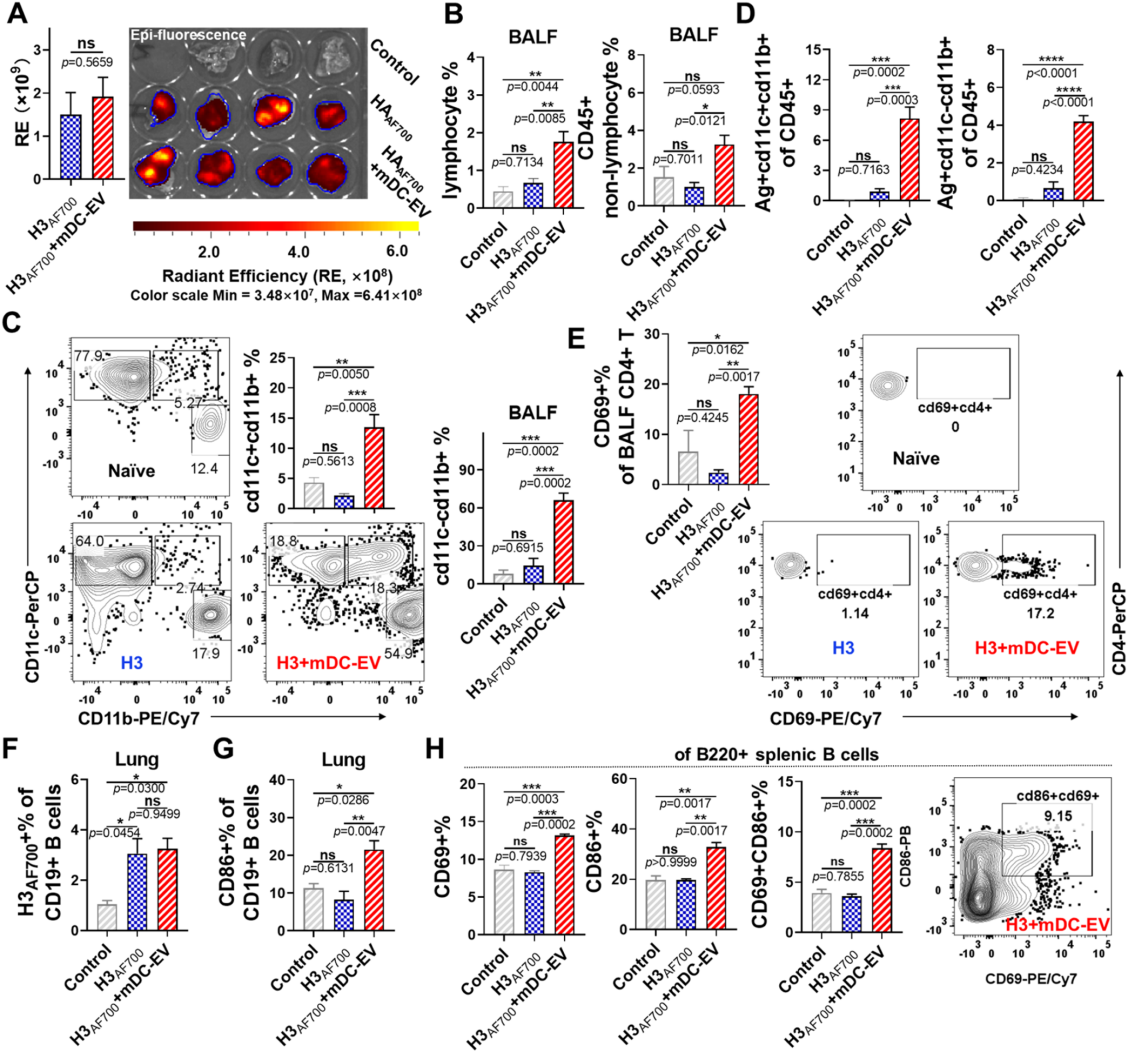
Initiation of early immune responses 1 day post-immunization. (A) Antigen (Ag, H3_AF700_) delivery to mouse lungs, recorded by an *in vivo* imaging system. Automatic ROI tools with a 6% threshold were used for ROI analysis. (B) The lymphocyte and CD45+ nonlymphocyte cell frequencies in BALF. (C) The recruited CD11c+CD11b+ and Ag+CD11c-CD11b+ cell frequencies among CD45+ non-lymphocytes. (D) The frequencies of Ag+CD11c+CD11b+ and Ag+CD11c-CD11b+ cells among CD45+ nonlymphocytes. (E) The CD69+ cell frequencies among CD4+ T cells in BALF. (F, G) The frequencies of antigen-positive and CD86+ cells in lung CD19+ B cells. (H) The frequencies of CD69+ and/or CD86+ cells among splenic B220+ B cells after incubation with H3_AF700_ or H3_AF700_+mDC-EVs for 24 h in vitro. Data are presented as mean ± SEM (*n* = 3 for the control group and *n* = 4 for immunization groups in A-G, *n* = 3 for H). Statistical significance was analyzed using Student’s *t*-test or one-way ANOVA followed by Tukey’s multiple comparison tests (*p* > 0.05, ns, not significant; *p < 0.05; **p < 0.01; ***p < 0.001; ****p < 0.0001).

Further analysis by flow cytometry shows that H3_AF700_+mDC-EVs vaccination substantially increased the numbers of mouse airway lymphocytes and CD45+ nonlymphocytes compared to the naïve and H3_AF700_ groups, suggesting enhanced immune cell recruitment (Figures 7B and S8A,B). Moreover, distinct nonlymphocyte subpopulations were observed based on the differentiation of CD11c and CD11b expression (Figures 7C and S8C). The H3_AF700_+mDC-EVs group recruited significantly higher CD11c+CD11b+ and CD11c-CD11b+ but lower CD11c+CD11b- populations than the other two groups. Further analysis revealed that the majority of CD11c+CD11b+ cells were CD103+CD11b+ cells, while most CD11c-CD11b+ cells were identified as Ly6C+ neutrophils or inflammatory monocytes, indicating the activation of innate immune responses (Figure S8D). Furthermore, H3_AF700_ antigens were internalized by these subpopulations without significant differences between the H3_AF700_ and H3_AF700_+mDC-EVs groups (Figure S8E). The H3_AF700_+mDC-EVs group displayed significantly higher frequencies of Ag+CD11c+CD11b+ and Ag+CD11c-CD11b+ cells, alongside lower frequencies of Ag+CD11c+CD11b- cells, likely due to the distinct cell subpopulation abundances (Figure 7D and S8F). Similarly, substantially boosted CD45+ nonlymphocytes (Figures S9) and total (Figures S10A,B) or Ag-internalized (Figure S10C) CD11c+CD11b+ and CD11c-CD11b+ subpopulations, but not CD11c+CD11b- subpopulations were observed in the lungs of mice from the H3_AF700_+mDC-EVs group. However, H3_AF700_ antigens were more readily internalized by CD11c+CD11b+ and CD11c-CD11b+ cells in the H3_AF700_+mDC-EVs group and by CD11c+CD11b- populations in the H3_AF700_ group (Figure S10D). Since dendritic cells are pivotal for antigen presentation and the initiation of adaptive immune responses, the distinct antigen cellular uptake observed in the H3_AF700_+mDC-EVs group likely underlies the resulting robust immune responses.

The recruitment of Ly6C+ neutrophils or inflammatory monocytes may indicate potential immune toxicity. To further assess the safety of the mDC-EVs, we evaluated pro-inflammatory cytokine levels in nasal washes and BALF 1 day post-immunization (Figure S10E). LPS (1 μg per mouse) was used as the positive control. No apparent IL-12 secretion was detected in both groups. However, the LPS group elicited significant IL-6 in mouse nasal washes and BALF. In contrast, although mDC-EVs induced slightly higher IL-6 levels than the naïve control group, no significant difference was observed, indicating favorable safety and biocompatibility.

We also evaluated early lymphocyte responses. H3_AF700_+mDC-EVs immunization significantly promoted enhanced early T cell activation in CD4+ and CD8+ airway and lung T cells, as suggested by the expression of CD69 (Figures 7E, S11 and 12). By contrast, H3_AF700_ alone did not show such effects. In addition, comparable amounts of H3_AF700_ antigens were observed to be internalized by lung CD19+ B cells in both groups, consistent with the IVIS results (Figure 7F). However, only the mDC-EVs adjuvanted group displayed apparent early B cell activation (Figure 7G). Expanded CD86+Ag+CD19+ B cells were observed from the H3_AF700_+mDC-EVs group compared to the H3_AF700_ group (Figure S12C). We assessed serum antibody responses at early time points. Consistently, the immune sera from the H3 + mDC-EVs group exhibited significantly elevated levels of antigen-specific IgG and IgA antibodies at 7 days post-immunization compared to the H3 group (Figure S12D). We further investigated whether mDC-EVs can directly influence antigen uptake in B cells and cell activation in vitro by culturing splenocytes. Adding mDC-EVs promoted the internalization of H3_AF700_ into B220+ B cells (Figure S13) and B cell activation, as indicated by the enhanced CD69 and CD86 expression (Figure 7H). Although H3_AF700_ was effectively internalized by B cells, no significant changes in CD86+, CD69+, and CD86+CD69+ frequencies were observed compared to the untreated control (Figure 7H).

Lastly, to study which components contribute to the adjuvanticity of mDC-EVs, the effects of mDC-EVs on immature BMDC were compared with those of mDC-extracted cell membrane proteins (mDC-mem) (Figure S14). mDC-mem and mDC-EVs displayed distinct effects, with mDC-mem only enhancing CD40 and CD86 but not CD80 expression (Figure S14B,C). Additionally, mDC-mem elicited the production and secretion of IL-6 but not IL-12 and TNF-α (Figure S14D). Therefore, the cell membrane component can only partially account for the robust adjuvanticity of mDC-EVs, and other elements specific to the mDC-EV surface and/or contained in the interior of mDC-EVs also play a role in their immunostimulatory effects.

### Germinal Center and T-Cell Responses in mLNs 14 Days Post-Immunization

Mediastinal lymph nodes (mLNs) are crucial for mucosal immune responses by serving as the primary site where immune cells encounter and respond to antigens from mucosal surfaces.^31,32^ T cell priming in mLNs and migration to lung tissues are essential for the optimal induction of mucosal sIgA and pulmonary T_RM_ induction.^31,32^ To better understand the underlying mechanisms of the enhanced mucosal immune response and protection in the H3+mDC-EVs group, we assessed germinal center (GC) and T-cell responses in the lung-draining mLNs 14 days post-immunization (Figure 8A).

**Figure 8:**
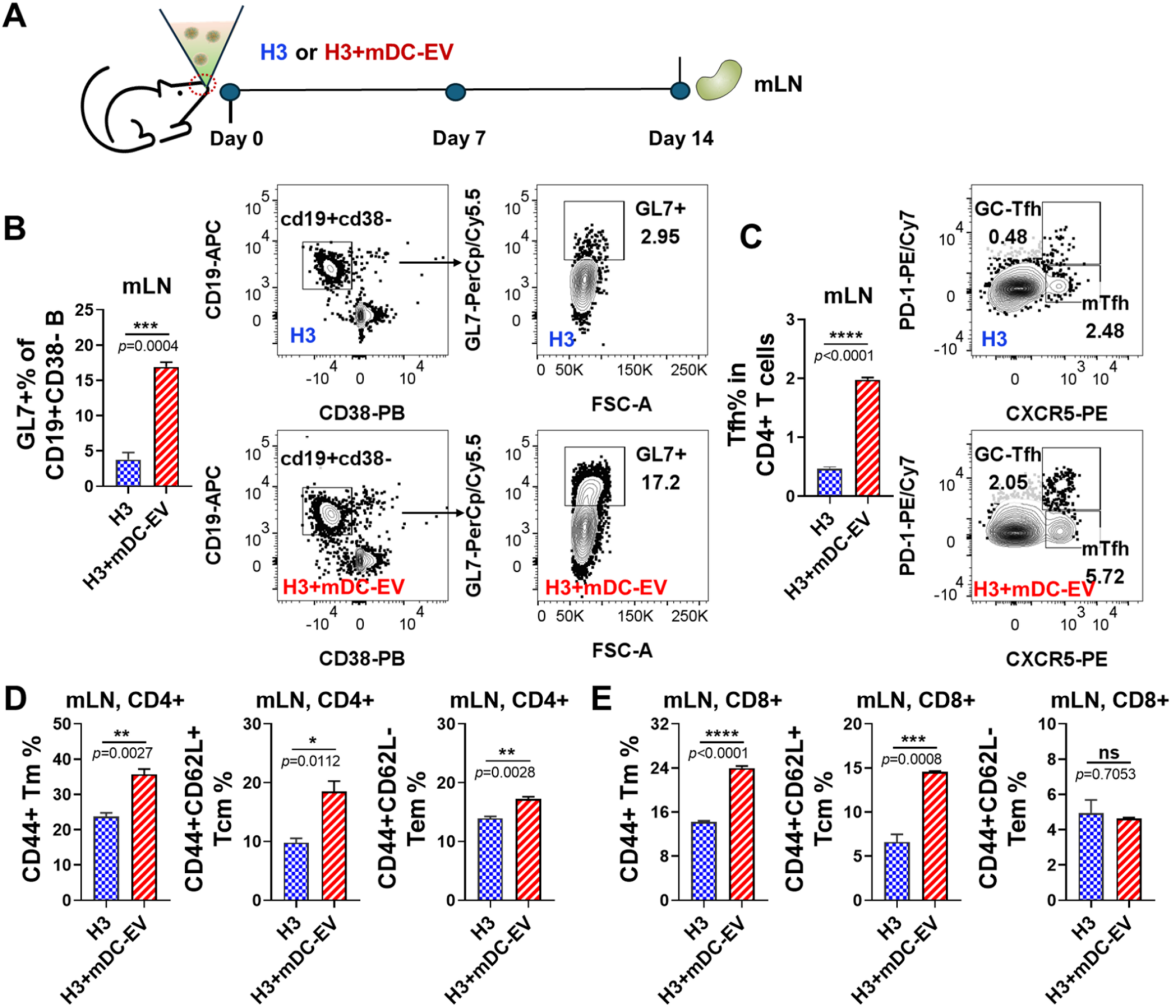
Germinal center and T-cell responses in mLNs of immunized mice 2 weeks post-immunization. (A) Schematic diagram of immunization and sample collection. (B) CD19+CD38-GL7+ GC B cell responses. (C) Tfh cell responses. The GC Tfh and mTfh cells were gated as CD4+CXCR5+PD1+ and CD4+CXCR5+PD1- cells, respectively. (D, E) The frequencies of antigen-experienced CD44+ Tm, CD44+CD62L+ Tcm, and CD44+CD62L- Tem cells within CD4+ and CD8+ T cell populations in mLNs. Data are presented as mean ± SEM (*n* = 3 mice per group). Statistical significance was analyzed using Student’s *t*-test (*p* > 0.05, ns, not significant; *p < 0.05; **p < 0.01; ***p < 0.001; ****p < 0.0001).

GC B and T follicular helper (Tfh) cells are crucial for eliciting a robust humoral immune response. Notably, immunization with H3+mDC-EVs elicited a significant increase in the populations of CD19+CD38-GL7+ GC B cells and CD4+CXCR5+PD1+ GC Tfh cells in the mLNs compared to the H3 group (Figures 8B,C). Furthermore, we evaluated the antigen-experienced (CD44+) memory T (Tm), CD44+CD62L+ central memory T (Tcm), and CD44+CD62L- effector memory T (Tem) cell populations in mLNs. A single-dose immunization with H3+mDC-EVs resulted in significantly increased populations of CD4+ Tm, Tcm, and Tem cells, as well as CD8+ Tm and Tcm cells, compared to the H3 group (Figure 8D,E). These findings revealed that H3+mDC-EVs adjuvantation efficiently enhanced the development of germinal center reactions and memory T cell responses in lung-draining lymph nodes, thereby contributing to the induction of robust localized adaptive immune responses within the respiratory tracts.

## Discussion

Developing safe and potent mucosal adjuvants is crucial for applying protein or peptide subunit vaccines in mucosal routes. Influenza HA is weakly immunogenic and cannot confer complete protection when administered intranasally.^21,33^ Herein, we show that mDC-EVs, rather than imDC-EVs, can function as a potent mucosal adjuvant for protein-based HA subunit vaccines. Adjuvanted HA immunization with mDC-EVs boosted robust antigen-specific systemic and mucosal immune responses, including balanced Th1- and Th2-type antibodies, antigen-specific IgG- and IgA-secreting cells, IL-2-, IL-4-, and IFN-γ-secreting memory T cells, as well as robust mucosal sIgA and local cellular immune responses. These immune responses provided potent protection against homologous Aic virus challenges in immunized mice.

Influenza is a highly variable virus, necessitating regular updates to the vaccine. To address this problem, universal influenza vaccines that elicit cross-reactive and protective immune responses are urgently needed. Our results demonstrated that mDC-EVs adjuvanted HA immunization could elicit multiple potential correlates of cross-protection against influenza,^34^ including broadly reactive IgG and sIgA antibodies to heterologous and heterosubtypic strains, cross-reactive systemic cellular immune responses, and local T_RM_ memory T cells, leading to enhanced cross-protection, as observed by the minor (<3%) body weight loss against 3 × LD_50_ heterologous Phi virus infection. Thus, mDC-EVs enhanced not only the magnitude but also the breadth of the induced immune response. Previously, we showed that 1 μg of CpG, a commonly used experimental intranasal adjuvant, adjuvanted HA (5 μg) immunization also protected mice from mortality; however, the mice suffered from more severe body weight loss (8 ∼ 12%) upon 2 × LD_50_ Phi infection.^21,22^ Thus, the nanoparticulate mDC-EVs adjuvant seemed to possess higher potency than the experimental CpG in enhancing cross-protection.

Targeting the conserved hemagglutinin stalk domain is one of the most promising approaches to developing universal influenza virus vaccines. Various strategies, such as chimeric HA vaccines or tailored HA stalk design, have been developed to elicit HA stalk-specific immune responses.^35,36^ However, these approaches involve complicated vaccine design, production, or purification processes. In this study, a simple H3+mDC-EVs mixture formulation effectively boosted substantial antibody and T cell immune responses against the head-removed HA stalk. By contrast, sole HA immunization failed to elicit such responses. Moreover, H3+mDC-EVs immunization can even elicit high levels of IgG antibodies to cross-group HAs, a phenomenon observed with a TLR7-nanoparticle adjuvant system in a recent study.^37^ Consistently, robust GC reactions, characterized by the expansion of GC B and Tfh cells, were detected 14 days postimmunization, which may facilitate the antibody diversification and affinity maturation.^38^ Tfh cells provide costimulatory signals and cytokines to GC B cells, facilitating class switch recombination, somatic hypermutation, and affinity maturation.^39^ The potent effect of mDC-EVs on promoting GC responses accounted for the increased breadth and cross-reactivity of antibody responses. With this, mDC-EVs adjuvanted HA vaccines can be a potential universal vaccine candidate in terms of immunogenicity and efficacy.

Our in vitro studies revealed that mDC-EVs, but not imDC-EVs, promoted dendritic cell maturation, cytokine secretion, inflammasome activation in macrophages, and B cell activation. This result is consistent with previous findings that mature DCs produced exosomes with the ability to elicit potent immune activation, whereas immature DC-derived exosomes did not.^40^ Consistently, imDC-EVs did not affect the HA’s immunogenicity or protection efficacy in mice. As EVs are membranous vesicles consisting of proteins, lipids, and nucleic acids, their composition and function are heterogeneous depending on physiological and pathological processes. Upon LPS stimulation, the mDCs produce distinct proteins, lipids, genetic materials, and other components that may be carried in the EVs, thereby contributing to their unique adjuvant effects.^41^ The molecular contents of EVs, such as miRNAs, likely determine their effects on inflammasome activation within target cells.^42^ In contrast, imDC-EVs are acknowledged to harbor anti-inflammatory properties and maintain immune tolerance,^41^ and thus fail to exhibit adjuvant activity for HA vaccines. Similarly, M1- but not M2-polarized macrophages secreted exosomes could potentiate cancer vaccinations by creating a pro-inflammatory microenvironment in the lymph node.^43^ Moreover, the possibility of LPS contamination was excluded in this study,^43^ highlighting the critical role of the EV itself.

Moreover, we demonstrated that the immunostimulatory effects of mDC-EVs were partially attributed to cell membrane structures. Cellular components inside EVs, such as miRNAs and cytokines, are other potential contributors to their adjuvanticity.^44^ Antigen delivery capability may also contribute to the adjuvant effects of EVs. 293T cell-derived EVs can deliver HAs through interactions between the HA head and sialic acid receptors present on EVs.^45^ However, it has been reported that the maturation of moDCs leads to a decrease in α-2,6-sialylation, which binds to human influenza virus HAs, and immature moDCs exhibit a high α-2,6-sialylation content.^46^ Therefore, the immunostimulatory functions, but not the delivery ability of HA, mainly account for the superior immunoenhancing effects of mDC-EVs. Without requiring specific antigen conjugation or antigen pulsing, this mDC-EV adjuvant system demonstrates high versatility and can be easily adapted for other protein antigens from various pathogens.

Further investigation of the cellular mechanisms revealed that H3+mDC-EVs immunization significantly enhanced the recruitment of both lymphocytes and nonlymphocytes to the mouse airways compared to H3 alone. After intranasal administration, most H3 antigens were internalized by airway and lung CD11c+ dendritic cells. Notably, despite comparable antigen delivery to the lung, H3+mDC-EVs-immunized mice showed unique antigen accumulation in CD11c+CD11b+CD103+ dendritic cells, in contrast to the H3-only group. Mucosal CD11b+CD103+ dendritic cells were reported to be essential for generating mucosal antibody responses and plasma cell pools in the bone marrow following flagellin immunization, as well as for efficiently inducing SM1 transgenic T cell activation.^47^ Given their prominent antigen uptake, these CD11b+CD103+ dendritic cells likely played a key role in antigen presentation and the initiation of robust adaptive immune responses in the H3+mDC-EVs group. Additionally, we observed expanded CD11c-CD11b+Ly6C+ neutrophils and inflammatory monocytes 1 day postimmunization, indicating effective induction of innate immune responses. Moreover, H3+mDC-EVs immunization triggered early activation of CD4+ and CD8+ T cells, as well as CD19+ B cells, in the airways and lungs, and strong central and effector memory T cell responses in mLNs, which also contributed to the adjuvanticity of mDC-EVs. Thus, the adjuvant activity of mDC-EVs is believed to occur through multiple signal pathways and mechanisms, including the distinct immune cell recruitment and uptake, DC maturation, inflammasome activation, lymphocyte activation, and the induction of germinal center reactions.

Developing safe and potent mucosal adjuvants to broaden immunity breadth and enhance mucosal immunity is a viable approach to achieving the goal of universal influenza vaccines. The inherent biocompatibility and potent adjuvant effects of mDC-EVs make them an attractive adjuvant for mucosal vaccine development. However, practical challenges, such as scalability, would arise in translating mDC-EV adjuvants for clinical use, necessitating more advanced, easily manipulated, and scalable dendritic cell culture and EV production techniques. Their long-term safety and stability also require further investigation. Nonetheless, our findings provide valuable insights into the in vivo role of mDC-EVs and their potential in advancing vesicle-based mucosal immunotherapies and vaccines. In addition to employing mDC-EV directly as adjuvants, exploring how existing commercial vaccine adjuvants influence the production of EVs and their potential role in adjuvanticity in human populations would also be an intriguing topic. Developing vaccine or adjuvant formulations that promote the specific EV production in immunized hosts might also be a practical strategy to harness their robust adjuvanticity.

## Conclusions

In summary, this study demonstrates that mature BMDC-derived mDC-EVs function as potent mucosal adjuvants for recombinant protein-based influenza HA vaccines. These vesicles exhibit intriguing immunostimulatory activity both in vitro and in vivo. Specifically, they effectively activated antigen-presenting dendritic cells, macrophages, and B cells in vitro, and promoted enhanced recruitment of airway immune cells, distinct cellular uptake patterns, early lymphocyte activation, and robust germinal center formation in female Balb/c mice. Intranasal immunization with HA+mDC-EVs elicited significant, cross-reactive, and multifaceted humoral and cellular immune responses at both systemic and mucosal sites, conferring complete protection against homologous and heterologous influenza virus challenges. Collectively, these findings underscore the potential of mDC-EVs as a promising adjuvant or immunomodulatory target for the development of mucosal vaccines.

## Methods

### Study Design

The primary objective of this study was to investigate whether EVs derived from dendritic cells can enhance the immunogenicity and cross-protection potency of recombinant influenza HA protein vaccines when administered intranasally. To this end, we examined the immunostimulatory effects of DC-EVs on antigen-presenting cells and their capability to boost cross-reactive antibody, cellular, and mucosal immune responses to intranasal Aichi H3 vaccines, using soluble H3 as a control. We further examined their prophylactic protection efficiencies against homologous and heterologous influenza virus challenges and the cellular mechanisms underlying the adjuvanticity of mDC-EVs in mice. The number of mice per experimental group is specified in the figure legends and methods. Statistical analyses were conducted when applicable.

### Ethics Statement

Female BALB/c mice (6–8 weeks old) purchased from Envigo were used in this study. The mice were housed at the Georgia State University animal facility under 12-h light/12-h dark cycles, with temperatures ranging from 20 to 23 °C, and a relative humidity of 45–55%. This study was approved by the Georgia State University Institutional Animal Care and Use Committee (IACUC). All animal experiments were conducted following the IACUC guidelines of Georgia State University under IACUC protocol A22029. Mice were adapted for at least 1 week before the experiments, and then randomly assigned to experimental groups.

### Proteins and Viruses

The recombinant trimetric influenza HA ectodomains, A/Aichi/2/1968 (Aic, H3N2) H3 (HA gene from GenBank No: CY121117.1), A/mallard/Netherlands/1/1999 (Net, H4N6) H4 (full-length HA plasmids from BEI resources, Catalog No.: NR-28996), and A/mallard/Sweden/51/2002 (Swe, H10N2) H10 (full-length HA plasmids from BEI resources, Catalog No.: NR-29002), A/PR8/8/1934 (PR8, H1N1) H1 (HA gene from GenBank No:), A/mallard/Sweden/86/2003 (Swe, H12N5) H12 (full-length HA plasmids from BEI resources, Catalog No.: NR-29004), and A/Eurasian wigeon/Netherlands/4/2005 (Net, H9N2) H9 (full-length HA plasmids from BEI resources, Catalog No.: NR-29001) were expressed and purified as previously described.^48^ Briefly, the Bac-to-Bac baculovirus expression system (Thermo Fisher Scientific) and 9 (Sf9, American Type Culture Collection (ATCC), CRL-1711) cells were used for protein expression. Sf9 cells were cultured in serum-free ESF 921 Insect Cell Culture Medium (Expression Systems). The HisPur Ni-NTA resins (Catalog No.: 88223, Thermo Scientific) were employed for protein purification. The concentration of purified proteins was determined by the Micro BCA Protein Assay Kit (Thermo Scientific). The protein purity was confirmed by reducing sodium dodecyl sulfate-polyacrylamide gel electrophoresis (SDS-PAGE) followed by Coomassie Blue staining. Recombinant A/Hong Kong/4801/2014 (HK, H3N2) HA (Catlog No.: NR-58621), A/Wisconsin/67/2005 (Wis, H3N2) HA (Catlog No.: NR-49237), A/Anhui/1/2013 (Anh, H7N9) H7 (Catlog No.: NR-44365 and NR-44081), and A/Singapore/1/1957 (H2N2) (Catalog No.: NR-52249) were ordered from BEI resources.

The propagation of Aic, Phi, Wis, and reassortant A/Shanghai/2/2013 (rSH, H7N9) influenza viruses was performed in embryonated chicken eggs as previously described.^49,50^ The Aic and Phi viruses used in mouse-challenging studies were mouse-adapted. The LD50, or median lethal dose, was determined using the Reed and Muench method in advance.

### Culture of BMDC and BMDM

Bone marrow cells were harvested from the bone marrow of 6–8-week-old naive female Balb/c mice and cultured as described previously.^51,52^ Briefly, bone marrow progenitors were extracted from isolated mouse femurs and tibias and cultured at a density of 2 × 10^6^ cells/mL in BMDC culture medium (RPMI 1640 supplemented with 2 mM l-glutamine, 10% fetal calf serum, 100 U/ml penicillin/100 μg/mL streptomycin, 20 mM HEPES, 1 × nonessential amino acids, 55 μM 2-mercaptoethanol, 1 mM sodium pyruvate) containing 20 ng/mL recombinant GM-CSF (Biolegend). Nonadherent cells were collected on day 3 and cultured in a new Petri dish, with medium refreshed every other day. On day 6, the suspending and loosely adherent cells were mainly immature BMDCs ready for use.

Bone marrow-derived macrophages (BMDMs) were cultured as previously described.^15^ Briefly, bone marrow progenitor cells are isolated and cultured at a density of 2 × 10^6^ cells/mL in BMDM culture medium (IMDM supplemented with 10% heat-inactivated fetal calf serum, 1× nonessential amino acids, and 100 U/ml penicillin/100 μg/mL streptomycin) containing 20 ng/mL macrophage colony-stimulating factor (M-CSF) in Petri dishes. An additional 5 mL of BMDM culture medium was added on days 3 and 5, respectively. On day 6, BMDM cells were ready for use.

### EV Isolations and Characterizations

BMDCs were cultured at 1 × 10^6^ cells/mL in BMDC culture media containing FCS depleted of bovine exosomes and protein aggregates (by ultracentrifugation at 100,000*g* for 2 h) for 2 days. The cells were left untreated for the collection of imDC-EVs or treated with 0.5–1 μg/mL of LPS for the collection of mDC-EVs. EVs were collected using the differential ultracentrifugation method. Briefly, cells were removed by centrifugation at 3000*g* for 20 min, and the culture medium was collected and centrifuged at 10,000*g* for 30 min to eliminate cell debris and large particles. The obtained supernatant was finally ultracentrifuged at 100,000*g* for 2 h at 4 °C to pellet down EVs. Purified exosomes were resuspended in sterile DPBS. The concentration of EVs was determined by measuring the protein content using Pierce Micro BCA Protein Assay Kits.

The hydrodynamic size and zeta potential of the obtained EVs were determined by dynamic light scattering using a Malvern Zetasizer Nano ZS (Malvern Panalytical). The EV morphology was recorded by Tescan Vega’s third generation scanning electron microscopy (SEM) (TESCAN Ltd., Czech Republic). The vesicles were fixed with 4% glutaraldehyde at 4 °C for 30 min before being deposited on a silicon wafer (Electron Microscopy Sciences, Hatfield, PA). After drying, the samples were coated with an additional thin layer of gold using a gold sputtering setup. Post-imaging adjustments were performed using the ImageJ software. The presence of EV marker proteins (CD81, TSG101, and Alix) on mDC-EVs and imDC-EVs was determined by Western blot, using anti-CD81 (Cat. No.: 104901), anti-TSG101 (Cat. No.: 934302), and anti-Alix monoclonal antibodies (Cat. No.: 634501) purchased from BioLegend. Considering that LPS was added in the cell culture to promote DC maturation and may result in LPS contamination, the endotoxin levels in the purified mDC-EVs and imDC-EVs were further determined by the Pierce chromogenic endotoxin quant kit assay (Thermo Scientific, Cat: A39552S).

### In Vitro Stimulation of Dendritic Cells

To study the immunostimulating effects on dendritic cells, immature BMDCs were harvested, counted, and seeded in a 24-well tissue culture plate (1 × 10^6^ cells/well), followed by treatment with 15 μg/mL of imDC-EVs, 15 μg/mL of mDC-EVs, or 1 μg/mL of LPS (positive control) for 16–18 h. Untreated cells were used as the negative control. The stimulated cells were pelleted down, washed with FACS buffer (PBS supplemented with 2% FCS), and then stained with an antibody cocktail containing Fc blocker (CD16/32), antimouse CD11c-APC, CD40-PE, CD80-FITC, and CD86-APC/Cy7 antibodies for 30 min at 4 °C. Cells were washed and fixed with 4% PFA for 15 min at room temperature. The expression of dendritic cell maturation markers (CD40, CD80, and CD86) was assessed by flow cytometry. The antibodies used and gating strategies are presented in Figure S2A and Table S1 for the different experimental sets. The supernatant was collected to determine the levels of secreted proinflammatory cytokines (IL-6, TNF-α, IL-12, and IL-1β) using cytokine enzyme-linked immunosorbent assays (ELISA).

The immunostimulatory effect of mDC-EVs was further compared to that of mDC cell membrane proteins (mDC-mem). mDC-mem was prepared using a plasma membrane protein extraction kit (Abcam, Cat. ab65400). Fifteen μg/mL of mDC-EVs or mDC-mem were incubated with immature BMDCs for 16–18 h. The cells were stained with an antibody cocktail containing Fc blocker (CD16/32), Zombie NIR, antimouse CD11c-APC, CD40-PE, CD80-FITC, and CD86-PE/Cy7 antibodies for 30 min at 4 °C, followed by flow cytometry analysis to determine the expression of maturation markers. The gating strategies are presented in Figure S14A. The levels of secreted IL-6, IL-12, and TNF-α in the supernatant were determined by cytokine ELISA.

JAWSII cells were seeded at a density of 2 × 10^5^ cells/well and incubated with 15 μg/mL of mDC-EVs, 5 μg/mL of H3, or a mixture of H3+mDC-EVs for 24 h. The cells were then collected and stained with Zombie-Aqua (Biolegend) and an antibody cocktail containing Fc blocker (CD16/32), antimouse CD11c-APC, and CD86-PE/Cy7 antibodies for 30 min at 4 °C. The expression of CD86 was assessed by flow cytometry. The gating strategy is displayed in Figure S2C.

### Inflammasome Activation of Macrophages

We evaluated the capability of EVs to induce inflammation activation in BMDMs and THP-1 macrophages. BMDM cells were plated in BMDM culture medium into 24-well plates (8 × 10^5^ cells per well) and cultured overnight at 37 °C in a CO2 incubator. The cells were washed with warm DPBS and then incubated in 500 μL of BMDM stimulation medium (DMEM supplemented with 10% heat-inactivated FCS and 100 U/ml penicillin/100 μg/mL streptomycin) for 2h before treatment with 15 μg/mL of imDC-EVs, mDC-EVs, or 2 μg/mL of LPS for 16 h. Untreated cells were used as the negative control. LPS (2 μg/mL) plus ATP (5 mM, added for the last 30 min) was used as the positive control for inflammasome activation.

We evaluated the cleavage of pro-caspase-1 into mature caspase-1 in cell lysates, a key downstream event of inflammasome activation that facilitates the maturation of pro-interleukin (IL)-1β into IL-1β,^15,53^ and the IL-1β levels secreted in the cell cultures. Cells were lysed on ice using NP40 with protein inhibitors. After centrifuging to pellet the cell debris, the lysate supernatant was collected followed by Caspase-1 Western blotting with antimouse caspase-1 (p20) monoclonal antibody (AdipoGen, AG-20B-0042-C100) as the primary antibody. This antibody can recognize both full-length and activated (p20 fragment) mouse caspase-1. The supernatant was collected to determine IL-1β, IL-6, and IL-12 levels using cytokine ELISA.

Human monocytic THP-1 cells were cultured in RPMI 1640 medium supplemented with 10% fetal calf serum, 100 U/ml penicillin, 100 μg/mL streptomycin, and 0.05 mM 2-mercaptoethanol, as recommended on the ATCC Web site. Before the in vitro stimulation experiment, THP-1 cells were seeded in 96-well plates (2 × 10^5^ cells/well) and cultured in the presence of 100 mM phorbol,12- myristate,13-acetate (PMA) for 2 days to differentiate into THP-1 macrophages. The cell medium was removed and refreshed with RPMI 1640 medium without PMA. After a 2-day rest, the cells were treated with 15 μg/mL of imDC-EVs or mDC-EVs for 16 h. The supernatant was collected for analysis of IL-1β secretion by cytokine ELISA assay.

### Vaccination and Sample Collections

To compare the immunogenicity of H3+mDC-EVs versus H3, female Balb/c mice (6–8 weeks old, *n* = 3 per group) were intranasally immunized twice, with an 11-week interval, with 30 μL of H3 (3 μg)+mDC-EVs (18 μg) or H3 (3 μg) in DPBS (Figure 2A). Mouse body weight was monitored for 4 days postprime immunization. Immune sera were collected at 3 and 10 weeks postprime immunization (prime sera), and 3 weeks postboost immunization (boost sera), respectively, for antibody evaluation. Naïve mice sera were used as controls.

The immunized female Balb/c mice (6–8 weeks old, *n* = 3 per group) were euthanized 6 weeks postboosting immunization to evaluate the cellular and mucosal immune responses. Spleens, bone marrow, nasal washes, BALF, and lung tissues were collected. The spleens, bone marrows, and lungs were processed into single-cell suspensions followed by treatment with RBC Lysing Buffer Hybri-Max (Sigma-Aldrich) to remove the red blood cells. Mouse nasal washes and BALF were collected within 200 μL and 1.5 mL of ice-cold PBS supplemented with 0.5% BSA, respectively, and centrifuged at 550 g for 5 min to remove the cells. The resulting supernatant was stored at −20 °C for mucosal antibody analysis. The cells pelleted from BALF were treated with RBC Lysing Buffer Hybri-Max and subsequently resuspended in complete RPMI media for flow cytometry analysis.

### Challenge Studies

To study the protection efficacy of H3+mDC-EVs, H3+imDC-EVs, and H3 alone, female Balb/c mice (6–8 weeks, *n* = 5 per group) were immunized twice with H3+mDC-EVs (4/24 μg), or H3+imDC-EVs (4/24 μg), or H3 (4 μg) in DPBS at an interval of 4 weeks. Then, the immunized mice were intranasally challenged with 5 × LD_50_ of mouse-adapted homologous Aichi viruses or 2 × LD_50_ mouse-adapted heterologous A/Philippines/2/1982 (Phi, H3N2) at 4 weeks post-boosting immunization. Mouse body weights were monitored daily for 14 days postchallenge. A weight loss of >20% was used as a humane endpoint. The body weight area under the curve (AUC) for each group was calculated from the body weight curve by GraphPad Prism v8.0.

### Enzyme-Linked Immunosorbent Assay (ELISA)

Antigen-specific antibody responses in immune sera and mucosal washes were evaluated using the ELISA assay as previously described.^48^ Briefly, Serial dilutions of immune serum, nasal washes, or BALF were added to the 96-well ELISA plates (Thermo Scientific) precoated with proteins or formalin-inactivated viruses (4 μg per well, overnight at 4 °C) and incubated at 37 °C for 2 h. After washing, horseradish peroxidase (HRP)-conjugated goat antimouse IgG, IgG1, IgG2a, or IgA (SouthernBiotech) was added and incubated at 37 °C for 1 h. The plates were washed, and the chromogenic substrate, 3,3′,5,5′-tetramethylbenzidine was added. 1 M H2SO4 was used as the stop solution. The results (absorbance values at 450 nm) were recorded using a Biotek Epoch Microplate Reader (Agilent). The highest sample dilutions with an optical density at 450 nm (OD450) twice that of the naive control were considered the end point antibody titers. The end point IgG2b titers were calculated from the trendline equations using Microsoft Excel. Relevant antibody information is provided in Table S2.

H3 and homologous Aic virus were used as coating antigens to study the vaccine immunogenicity. Heterologous Phi H3N2, A/Wisconsin/15/2009 (Wis, H3N2), and heterosubtypic rSH H7N9, and A/Anhui/1/2013 HA (Anh H7, NR-44081 from BEI resources), and the conserved head-removed Aichi HA stalk (hrHA3) were used as coating antigens to evaluate the production of cross-reactive antibodies. sIgA levels in nasal washes and BALF were determined to assess the mucosal antibody responses.

### Serum Microneutralization Assay

A microneutralization assay was performed using Madin–Darby Canine Kidney (MDCK, NBL-2, ATCC CCL-34) cells to evaluate the cross-neutralization activity of the immune sera against heterologous Phi and heterosubtypic rSH virus strains, as previously described.^48^ The median tissue culture infectious dose (TCID50) of viruses was determined by the Reed and Muench method. In brief, 2-fold serial dilutions of receptor-destroying enzyme-treated and heat-inactivated immune sera were mixed with 100 TCID50 of viruses in the presence of TBCK-trypsin for 1 h at 37 °C, followed by overnight incubation with MDCK cells. An influenza nucleoprotein-based ELISA assay was performed to determine virus inhibition. Immune sera from 5 mice were pooled, and triplicate samples were tested.

### Enzyme-Linked Immunospot (ELISpot) Assay

ELISpot assays were performed to evaluate antigen-specific antibody (IgG, IgG1, IgG2a, and IgA)-secreting cell (ASC) frequencies and cytokine (IL-2, IL-4, and IFN-γ)-secreting cell frequencies in the mouse spleen collected 6 weeks post-boosting immunization. Relevant antibody information is provided in Table S2.

For the B cell ELISpot assay, sterile 96-well ELISpot filtration plates (Millipore) were precoated with purified H3 (200 μg/well) at 4 °C overnight. The plates were washed with PBS, blocked with culture medium for 2 h at 37 °C, and then splenocytes or bone marrow cells were seeded (5 × 10^5^ cells/well) and incubated at 37 °C for 16 h. After intensive washing to remove cells, 50 μL of HRP-conjugated goat antimouse IgG, IgG1, IgG2a, and IgA antibodies were added per well and incubated for 1 h at room temperature. Following intensive washes, KPL True Blue Peroxidase substrate (SeraCare) was added to develop spots. The reaction was terminated with rinsing water. Results were recorded with BIOSYS Bioreader-6000-E (BioSystem).

For the T cell ELISpot assay, 96-well ELISpot filtration plates were precoated with antimouse IL-2, IFN-γ, or IL-4 capture antibodies (200 μg/well) at 4 °C overnight, followed by washing and blocking. Splenocytes were seeded at 5 × 10^5^ cells/well and incubated with 4 μg/mL of H3 for 2 days. After intensive washing to remove the cells, 50 μL of biotin-conjugated antimouse IL-2, IFN-γ, or IL-4 detection antibodies were added per well and incubated for 1 h. After washing, streptavidin-HRP (Cat. No.: 554066, BD Biosciences) was added for another 1 h incubation. The spot development, reaction termination, and data recording procedures are the same as those used in B-cell ELISpot assays.

### Local T Cell Responses Postboost Immunization by Flow Cytometry

We performed flow cytometry to evaluate the lymphocyte populations in the BALF and lungs of immunized mice collected 6 weeks post-boosting immunization. Cells were stained as detailed below, followed by fixation with 4% paraformaldehyde for 15 min at 4 °C before analysis. Results were recorded by a BD LSRFortessa Cell Analyzer (BD Biosciences), and data were analyzed using the FlowJo v.10 software.

For BALF T cell analysis, cells in the BALF were pelleted by centrifugation at 550g for 5 min, washed with FACS buffer and stained with an antibody cocktail containing Zombie NIR viability dye, antimouse CD4-Percp/Cy5.5, CD8α-FITC, CD44-BV421, CD69-PE/Cy7, CD49a-PE, and CD16/32 antibodies for 30 min at 4 °C. The gating strategy for the T cell populations is displayed in Figure S6A.

For lung T cell analysis, isolated mouse lungs were minced into small pieces and incubated with digestion medium (RPMI 1640 media supplemented with 1 mg/mL Collagenase D (Roche) and 30 μg/mL DNase I (Sigma-Aldrich)) at 37 °C for 35–40 min. The tissues were then processed into a single-cell suspension by grinding them through 70 μm cell strainers (Fisherbrand, Cat. No. 22363548). Cells were treated with RBC Lysing Buffer Hybri-Max to remove red blood cells and washed with FACS buffer. One-third of the cells were stained with the lung T cell antibody cocktail containing Zombie NIR viability dye, antimouse CD4-Percp/Cy5.5, CD8α-FITC, CD44-BV421, and CD16/32 antibodies for 30 min at 4 °C. Antigen-experienced T cell populations were gated as described in Figure S6B.

### Antigen Delivery and Early Immune Responses

H3 was fluorescently labeled with Alexa Fluor 700 NHS Ester (Succinimidyl Ester) using the NHS amidation reactions. To evaluate the antigen delivery profile and early immune responses postimmunization, we immunized female Balb/c mice (*n* = 3–4) with H3_AF700_ (3 μg per mouse) with or without mDC-EVs (18 μg per mouse). We collected sera, nasal washes, BALF, and lungs 24 h later. An in vivo imaging system (IVIS) was employed to evaluate the delivery of fluorescent H3AF700 antigens to the whole lungs. Images were recorded using a Caliper Life Sciences IVIS Spectrum system. The fluorescent intensity (radiant efficiency) of antigens in the lungs was quantified by the Living Image software 4.8.2. Then, lung tissues were directly ground through 70 μm cell strainers to prepare single-cell suspensions. BALF cells were pelleted, and the supernatant was stored at −80 °C. The inflammatory cytokine levels in sera, nasal washes, and BALF supernatants were determined using cytokine ELISA assay.

To analyze the airway immune cell recruitment and antigen uptake in BALF and lung cells, cells were stained with an antibody cocktail containing Fc blocker (CD16/32), Zombie NIR viability dye, antimouse CD45-FITC, CD11c-PerCP, CD11b-PE/Cy7, and Ly6C–BV510 antibodies for 30 min at 4 °C, followed by fixation with 4% PFA, and recorded by flow cytometry. The subpopulation CD45+ cells with distinct CD11c and CD11b expression were gated out, and H3_AF700_ antigen uptake in these populations was analyzed. The gating strategies are presented in Figures S8A and 9A. To analyze the BALF T cell activation, cells were stained with an antibody cocktail containing Fc blocker (CD16/32), Zombie NIR viability dye, antimouse CD45-PE, CD4-PerCP, CD8-FITC, and CD69-PE/Cy7 antibodies. The gating strategies are presented in Figure S11A. To analyze antigen uptake in lung B cells and T cell activation, lung cells were stained with an antibody cocktail containing Fc blocker (CD16/32), Zombie NIR viability dye, antimouse CD45-PE, CD4-PerCP, CD8-FITC, CD69-PE/Cy7, CD19-APC, and CD86-PB antibodies. The gating strategies are presented in Figure S12A.

### In Vitro Stimulation of Splenic B Cells

To study whether the adjuvancation of mDC-EVs can directly influence antigen uptake in B cells and B cell activation, splenocytes were seeded in a 24-well tissue culture plate (1 × 10^6^ cells/well) and incubated with H3_AF700_ (4 μg/mL) with or without mDC-EVs (20 μg/mL) for 24 h. Untreated cells were used as the negative control. Cells were collected and stained with an antibody cocktail containing Fc blocker (CD16/32), antimouse CD11c-APC, CD4-PerCP, CD8-FITC, B220-PE, CD86-PB antibodies, and Zombie Aqua (Biolegend) for 30 min at 4 °C. The live CD4-CD8-CD11c-B220+ B cells were gated out, and H3_AF700_-positive and activated CD86-positive B cell populations were analyzed. The gating strategies are presented in Figure S13A.

### Germinal Center and Memory T Cell Response 14 Days Post-Immunization

To assess germinal center responses, mice were immunized once with H3 (4 μg per mouse), with or without mDC-EVs (24 μg per mouse), and euthanized 14 days later to collect mediastinal lymph nodes (mLNs). The mLN cells were analyzed using flow cytometry. The cells were divided into two parts and stained with different antibody cocktails. Antimouse CD19-APC, CD38-Pacific Blue, GL7-PerCP/Cy5.5, CD16/32 antibodies, and Zombie Aqua dye were used to detect GC B cells. For the detection of Tcm, Tem, and GC Tfh cells, antimouse CD4-PerCP, CXCR5-PE, PD-1-PE/Cy7, CD8-FITC, CD44-BV421, CD62L-APC/Cy7, CD16/32 antibodies, and Zombie Aqua dye were used. Cells were incubated with the respective antibody cocktails at 4 °C for 30 min, fixed, and recorded using the BD LSR Fortessa Cell Analyzer. The GC B cells were gated as CD19+CD38-GL7+ B cells. The GC Tfh cells were gated as CD4+CXCR5+PD1+ T cells. The mTfh cells were gated as CD4+CXCR5+PD1- T cells. The Tcm and Tem were gated as CD44+CD62L+ and CD44+CD62L- T cells, respectively.

### Statistical Analysis

Data are presented as mean ± SEM (standard error of the mean). Graphs were generated using GraphPad Prism v8.0 (GraphPad Software). Statistical analyses were performed using one-way analysis of variance (ANOVA), followed by Tukey’s or Dunnett’s multiple comparison tests or unpaired two-tailed Student’s *t* test, as indicated in the manuscript when applicable. The log-rank (Mantel-Cox) test was employed to compare survival curves between different groups. A probability value (*p*) of less than 0.05 is considered statistically significant. *p* > 0.05 is considered not significant (ns).

## Supplementary Material



## Notes

Additional in vitro characterization and stimulation experiment results of DC-EVs; mouse body weight changes postimmunization; additional systemic and mucosal antibody responses postimmunization; protection efficiencies of the H3 versus H3+ imDC-EVs; gating strategies for flow cytometry; antigen uptake behavior, and immune cell recruitment and activation postimmunization; comparison of the immunostimulatory effects of mDC-EVs versus mDC-mem; germinal center (GC) reactions; list of mouse antibodies (PDF)
