# Supplementary material for: Mature Dendritic Cell-Derived Extracellular Vesicles are Potent Mucosal Adjuvants for Influenza Hemagglutinin Vaccines
Source: ACS Nano. 2025 Jul 1;19(27):25526–42. doi: 10.1021/acsnano.5c08831 (PMC12269352; doi:10.1021/acsnano.5c08831)
Supplement: Supplementary file 1 [file nn5c08831_si_001.pdf]

Supplementary Information

Mature Dendritic Cell-Derived Extracellular Vesicles are Potent Mucosal Adjuvants for Influenza Hemagglutinin Vaccines

Chunhong Dong[1], Lai Wei[1], Wandi Zhu[1], Joo Kyung Kim[1], Ye Wang[1], Priscilla Omotara[1], Arini Arsana[1], Bao-Zhong Wang[1]

[1] Center for Inflammation, Immunity & Infection, Georgia State University Institute for Biomedical Sciences, 100 Piedmont Ave SE, Atlanta, Georgia 30303, USA

*Bao-Zhong Wang, Email: bwang23@gsu.edu

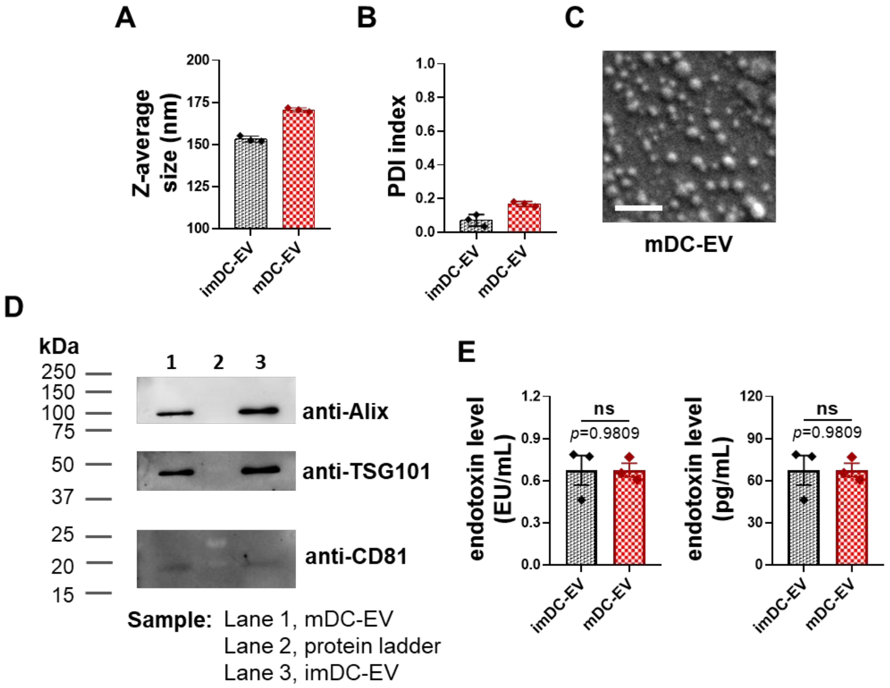

Fig. S1. In vitro characterization of DC-EVs. (A-B) Z-average size and the polydispersity index (PDI) of the obtained DC-EV particles, as determined by dynamic light scattering. (C) SEM image of mDC-EVs. The scale bar represents 500 nm. (D) Western blotting analysis of the EV surface biomarkers (Alix, TSG101, CD81) on mDC-EVs and imDC-EVs. (E) The endotoxin levels of the mDC-EVs and imDC-EVs suspensions (200 µg/mL, determined by BCA assay). The average endotoxin levels were 0.672 and 0.676 EU/mL for mDC-EVs and imDC-EVs, respectively, which equals around 67.2 and 67.6 pg/mL (Fig. S1E), indicating that one µL of EVs (around 200 ng, determined by BCA assay) contains less than 0.068 pg of endotoxin. Meanwhile, no significant difference was observed between mDC-EVs and imDC-EVs. Data are presented as mean ± SEM (n = 3 for A-B and E). Statistical significance was analyzed by Student's *t*-test in E ($p > 0.05$, ns, not significant).

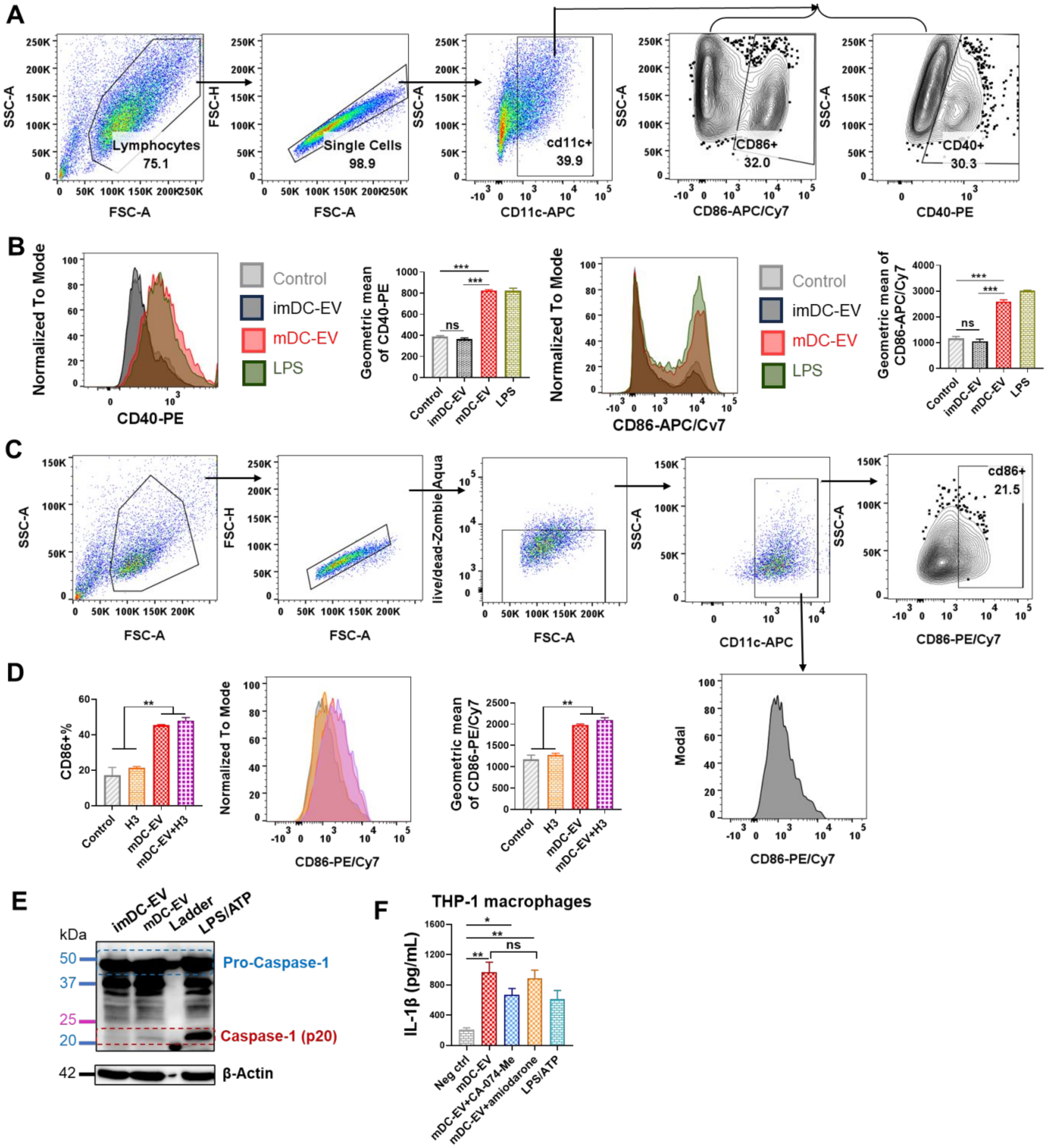

Fig. S2. In vitro stimulation experiment results. (A) Gating strategy of BMDC cells. (B) CD40 and CD86 expression on cultured BMDC cells by flow cytometry. (C) Gating strategy of JAWSII cells. (D) CD86 expression on cultured JAWSII cells by flow cytometry. (E) Caspase-1 activation in the DC-EVs-treated BMDM cell lysate. (F) The secreted human IL-1β levels in THP-1 macrophage cell cultures in the presence of inhibitors, CA-074-Me or amiodarone. Data are presented as mean ± SEM (n = 2 for B and D; n = 3 for F). Statistical significance was analyzed by one-way ANOVA followed by Tukey's multiple comparison tests ($p > 0.05$, ns, not significant; *$p < 0.05$; **$p < 0.01$; ***$p < 0.001$; ****$p < 0.0001$).

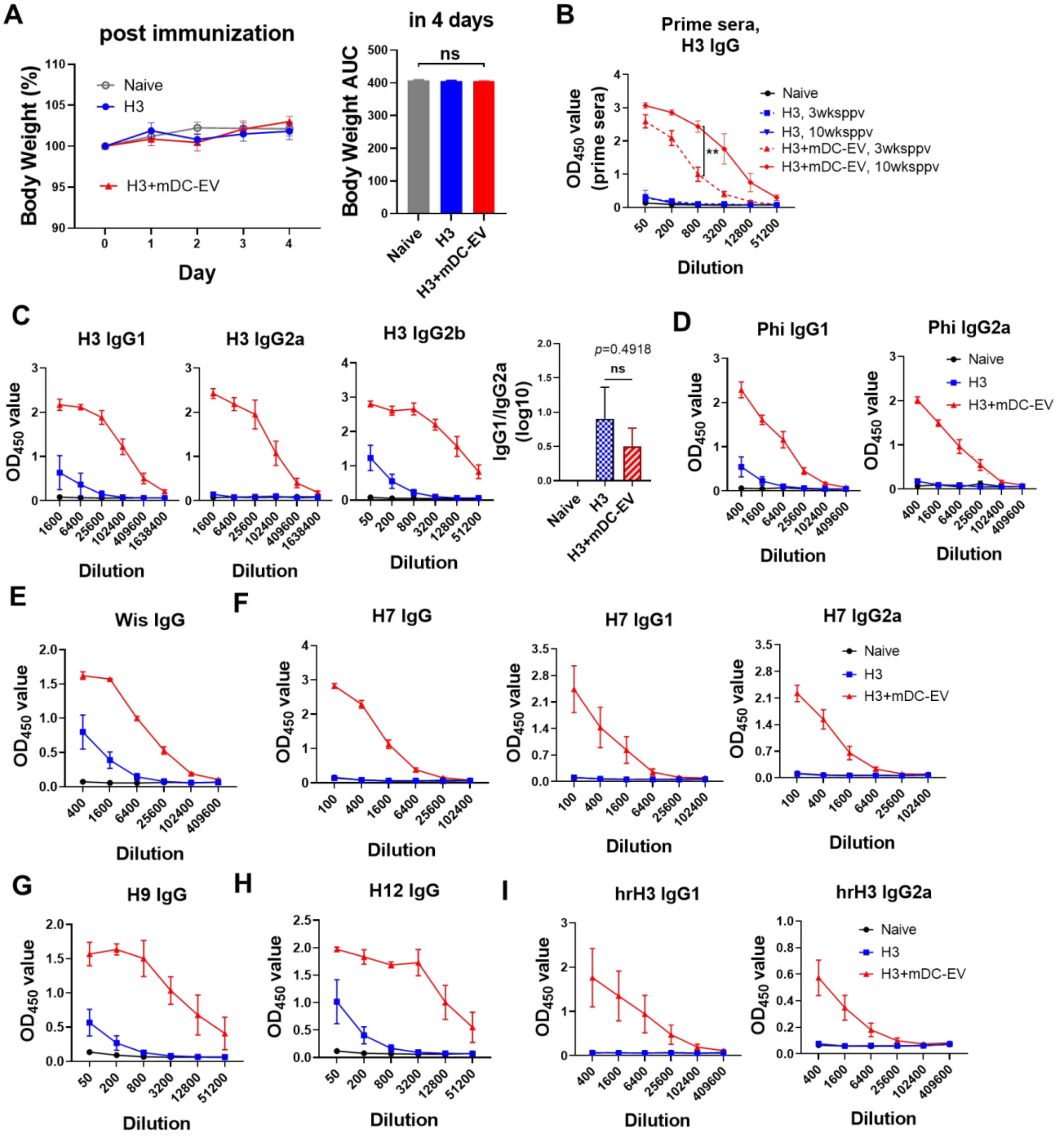

Fig. S3. Mouse body weight changes and serum antibody responses post-immunization. (A) Mouse body weight changes and body weight area under the curve (AUC) post-prime immunization. (B) H3-specific IgG levels in prime immune sera. (C) H3-specific IgG1, IgG2a, and IgG2b antibody levels, and the IgG1/IgG2a ratios in mice boost sera. The endpoint IgG2b titers were calculated from the trendline equations using Microsoft Excel. (D) Phi-specific IgG1 and IgG2a titers in boost sera. (E) Wis virus-specific IgG levels in boost sera. (F) Anh H7-specific IgG, IgG1, and IgG2a titers in boost sera. (G-H) IgG antibody levels against heterosubtypic H9 and H12. (I) hrH3-specific IgG1 and IgG2a titers in boost sera. Data are presented as mean ± SEM ($n = 5$ for A; $n = 3$ for B-I). Statistical significance was analyzed by Student's *t*-test in B (**$p < 0.01$).

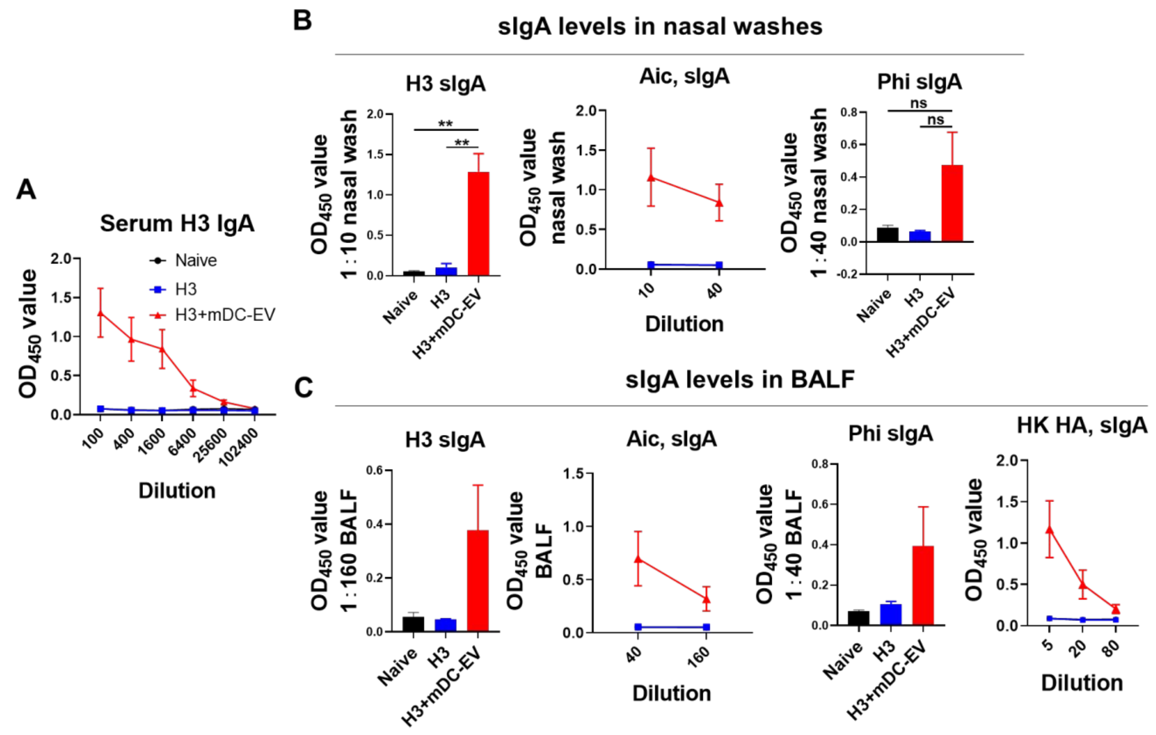

Fig. S4. IgA levels in immune sera and mucosal washes. **(**A) H3-specific IgA levels in boost immune serum. (B) sIgA antibody levels against H3, Aic, and Phi in nasal washes. (C) sIgA antibody levels against H3, Aic, Phi, and HK HA in BALF. Nasal washes and BALF were collected 6 weeks post-boosting immunization. Data are presented as mean ± SEM (n = 3 mice per group). Statistical significance was analyzed by one-way ANOVA followed by Tukey's multiple comparison tests ($p > 0.05$, ns, not significant; $*p < 0.05$; $**p < 0.01$; $***p < 0.001$; $****p < 0.0001$).

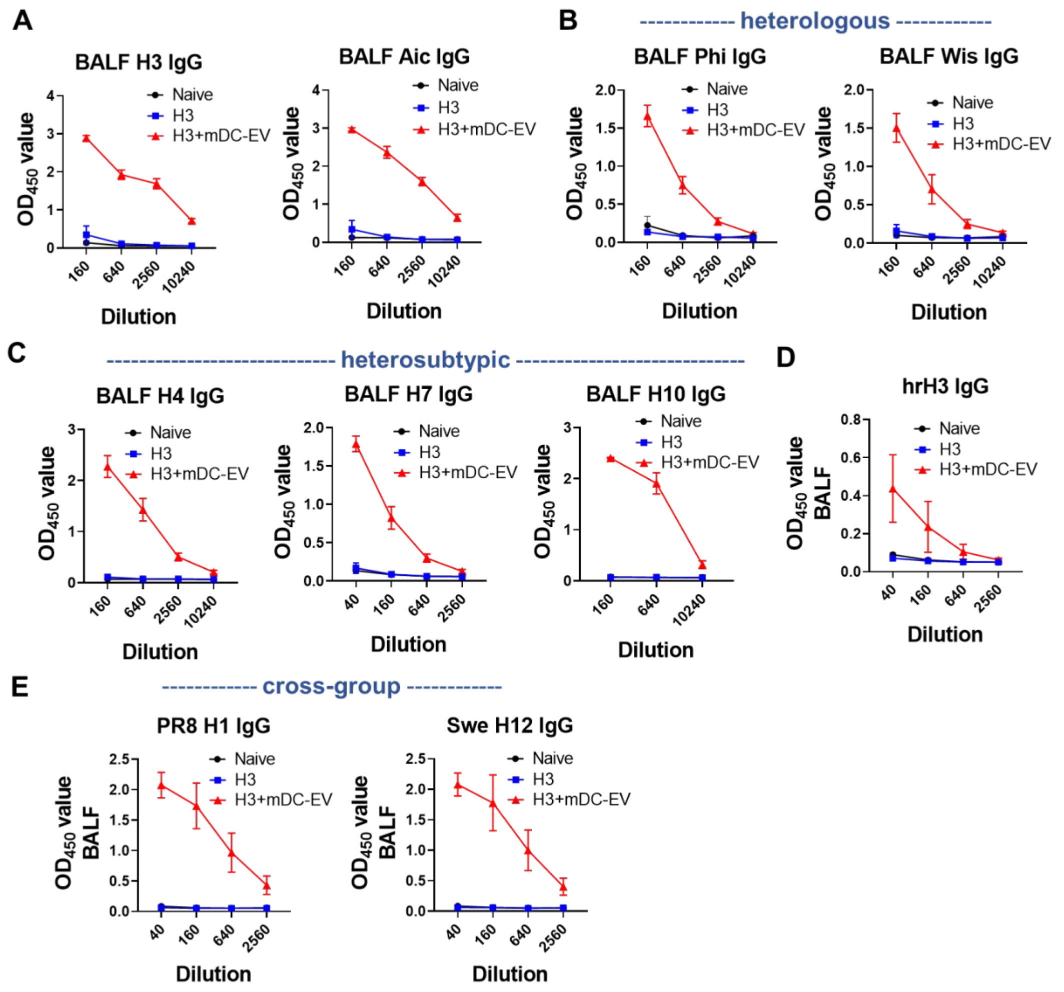

Fig. S5. Cross-reactive IgG antibody levels in BALF. (A) IgG levels against H3 and homologous Aic virus. (B) IgG levels against heterologous Phi and Wis viruses. (C) IgG levels against heterosubtypic Net H4, Anh H7, and Swe H10. (D) IgG levels against hrH3. (E) IgG levels against cross-group PR8 H1 and Swe H12. BALF was collected 6 weeks post-boosting immunization. Data are presented as mean ± SEM (n = 3 mice per group).

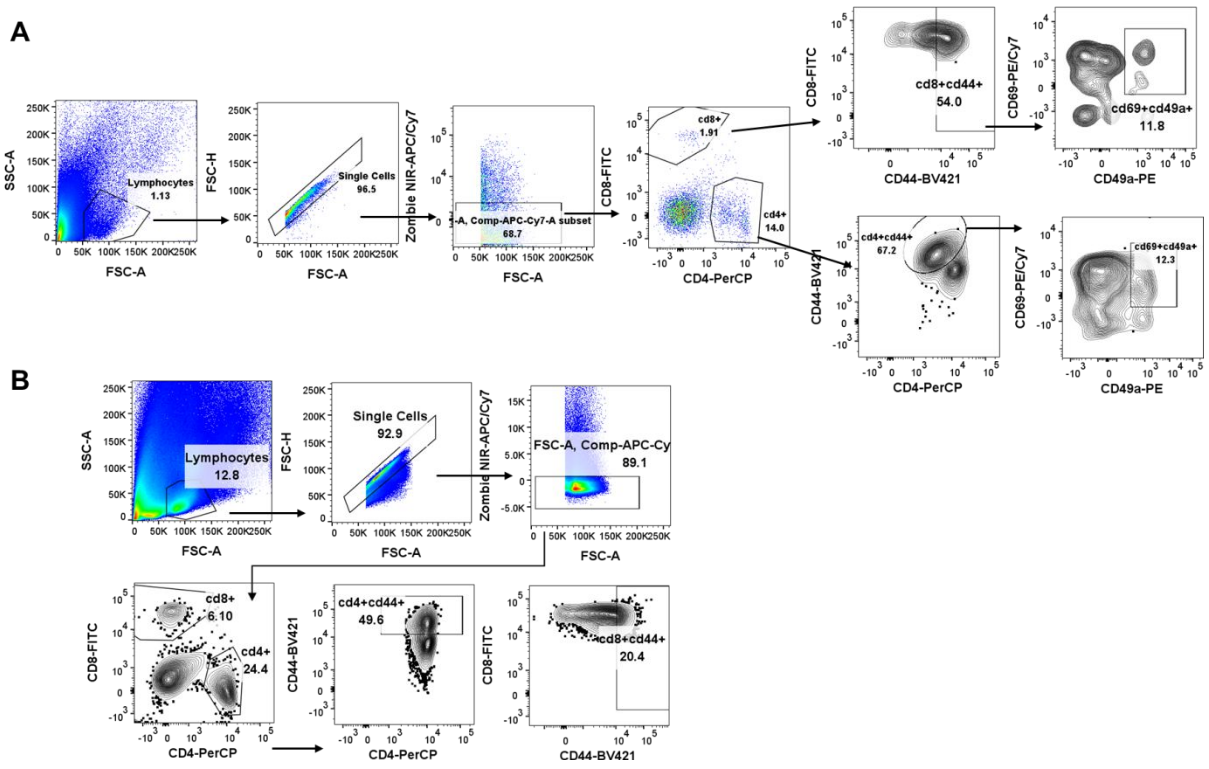

Fig. S6. Gating strategy for airway and lung T cell populations. An H3+mDC-EV-immunized mouse lung sample was used as an example. BALF and lung tissues were collected 6 weeks post-boosting immunization.

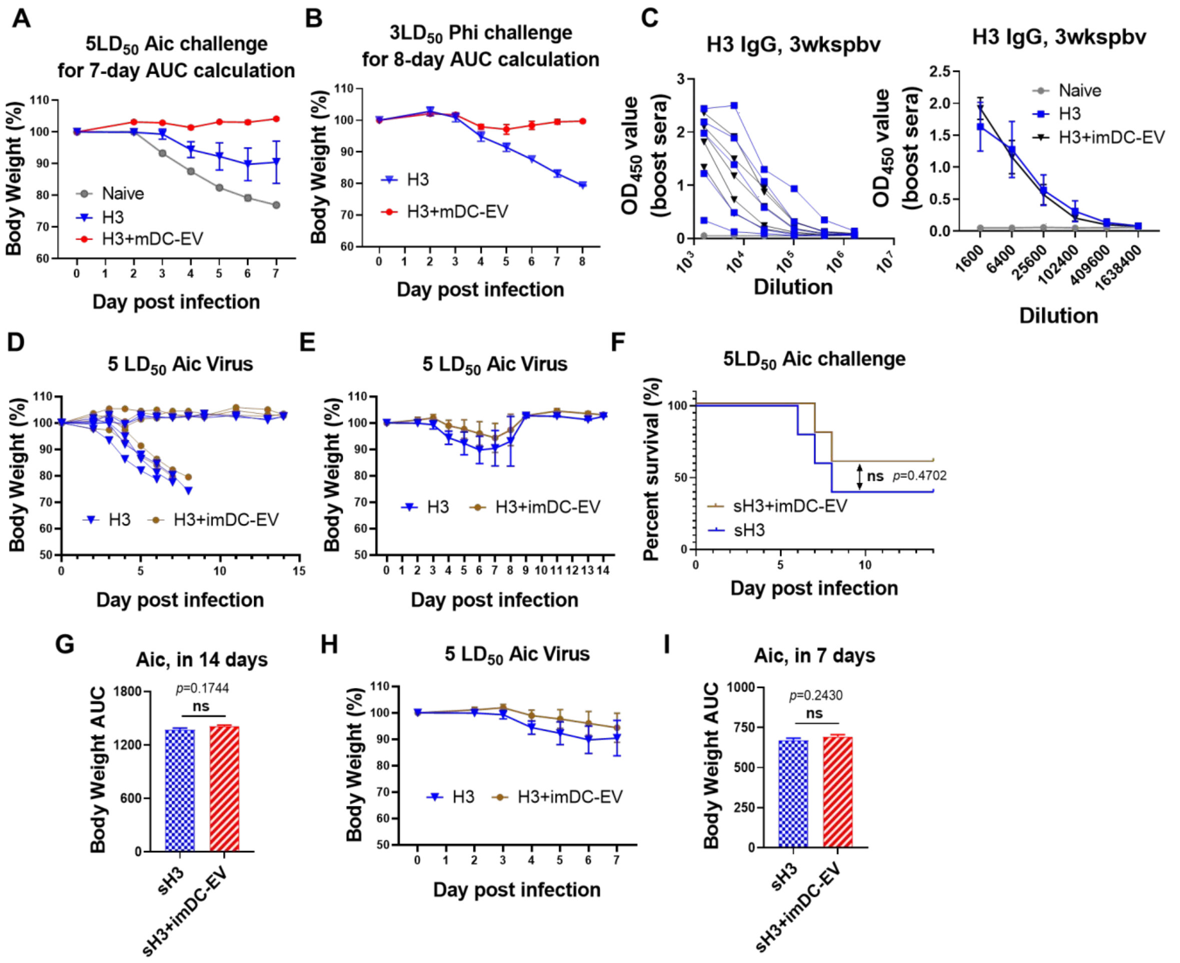

Fig. S7. Protection efficiencies upon homologous and heterologous influenza challenges. (A) Body weight curves for calculating the 7-day AUC post-Aic challenge. (B) Body weight curves for calculating the 8-day AUC post-Phi challenge. Data are presented as mean ± SEM (n=5 mice per group). (C) The comparison of antibody levels post-immunization. (D-E) The comparison of body weight changes between sH3 and sH3+imDC-Ev groups post 5LD50 Aic challenge. (F) The comparison of survival rates. (G) 14-day body weight AUC post-Aic challenge between the sH3 and sH3+imDC-Ev groups. (H) The body weight curves for calculating the 7-day AUC in I. (I) 7-day body weight AUC post-Aic challenge between the sH3 and sH3+imDC-Ev groups. Data are presented as mean ± SEM (n=5 mice per group). Statistical significance was analyzed by Student's *t*-test for G and I, or Log-rank (Mantel-Cox) test for F ($p > 0.05$, ns, not significant; $^{*}p < 0.05$; $^{**}p < 0.01$; $^{***}p < 0.001$; $^{****}p < 0.0001$).

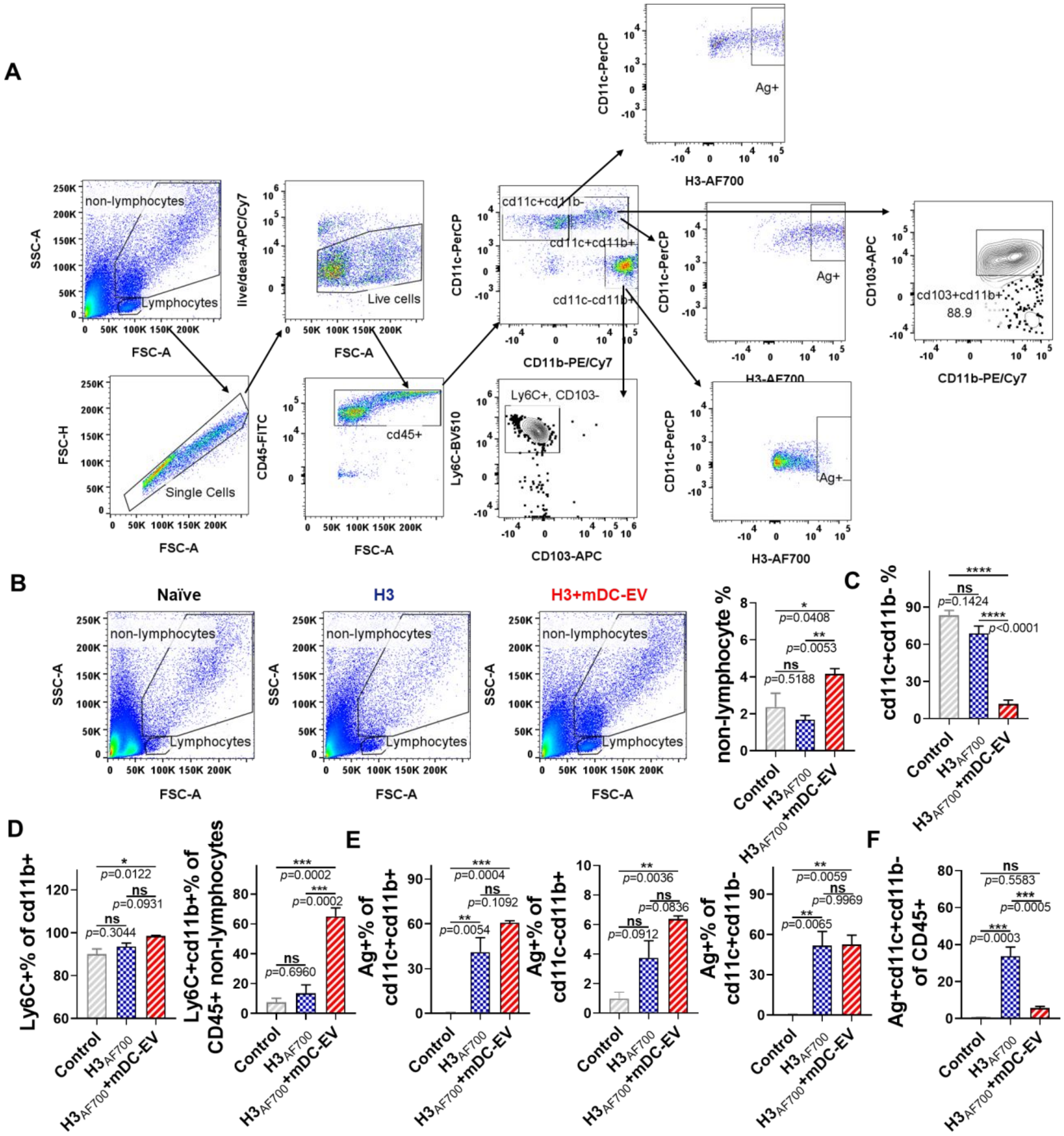

Fig. S8. Comparison of the non-lymphocyte immune cell subpopulations and antigen uptake behavior in BALF cells one day post-immunization. (A) Gating strategy. (B) Comparison of the lymphocyte and non-lymphocyte populations among groups. An H3+mDC-EV-immunized mouse lung sample was used as an example. (C) Comparison of the frequencies of CD11c+CD11b- cells. (D) Frequencies of Ly6C+ cells among CD11c-CD11b+, and Ly6C+CD11c-CD11b+ cells in CD45+ non-lymphocytes. (E) Frequencies of antigen (Ag, $H3_{AF700}$)-positive cells in BALF CD11c+CD11b-, CD11c+CD11b+, and CD11c-CD11b+ subpopulations. (F) Frequencies of Ag+CD11c+CD11b- cells in CD45+ non-lymphocytes. Data are presented as mean ± SEM (n = 3 for the control group and n = 4 for immunization groups). Statistical significance was analyzed by one-way ANOVA followed by Tukey's multiple comparison tests (p > 0.05, ns, not significant; *p < 0.05; **p < 0.01; ***p < 0.001; ****p < 0.0001).

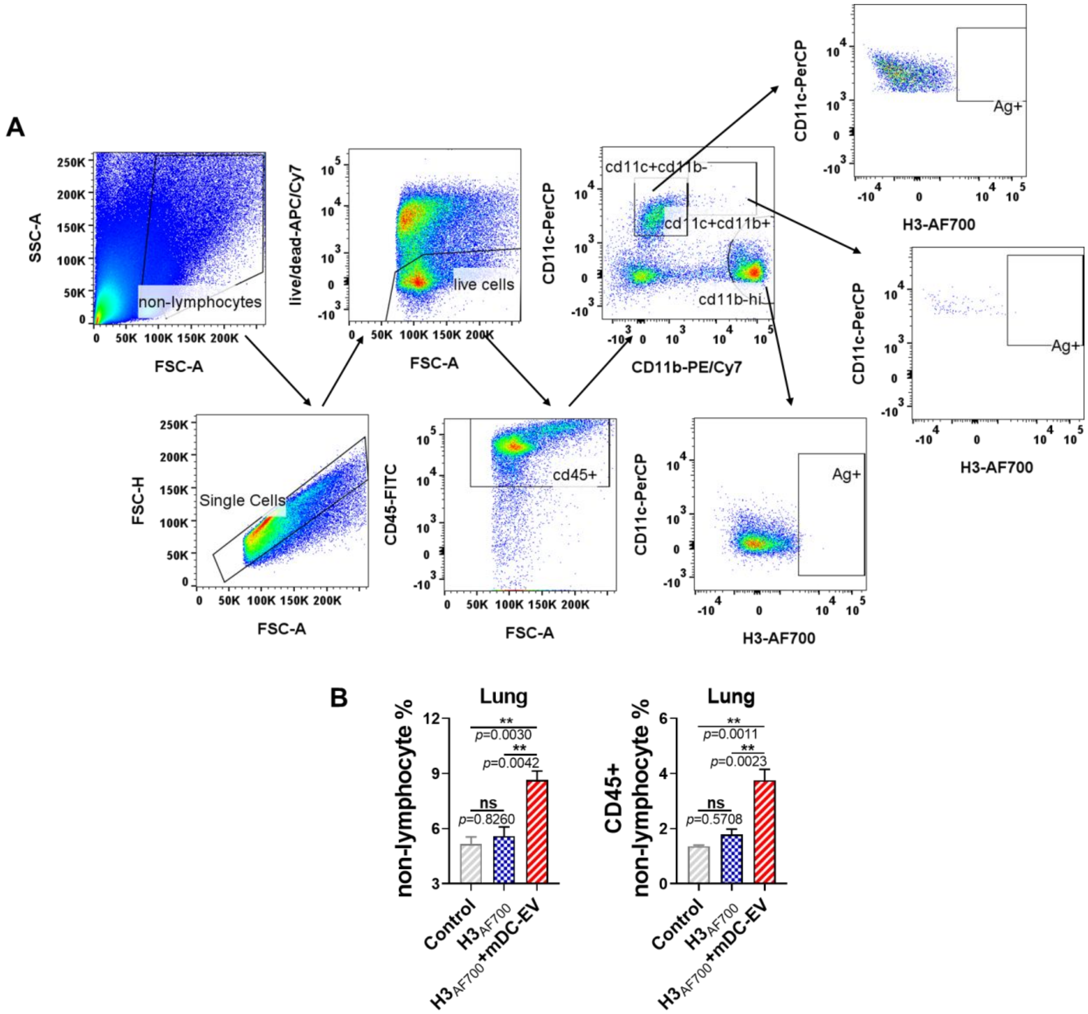

Fig. S9. Evaluation of non-lymphocyte subpopulations in lung cells one day post-immunization. (A) Gating strategy. A naïve mouse lung sample was used as an example. (B) Comparison of the frequencies of non-lymphocytes and CD45+ non-lymphocytes among groups. Data are presented as mean ± SEM (n = 3 for the control group and n = 4 for immunization groups). Statistical significance was analyzed by one-way ANOVA followed by Tukey's multiple comparison tests ($p > 0.05$, ns, not significant; $^{**}p < 0.01$).

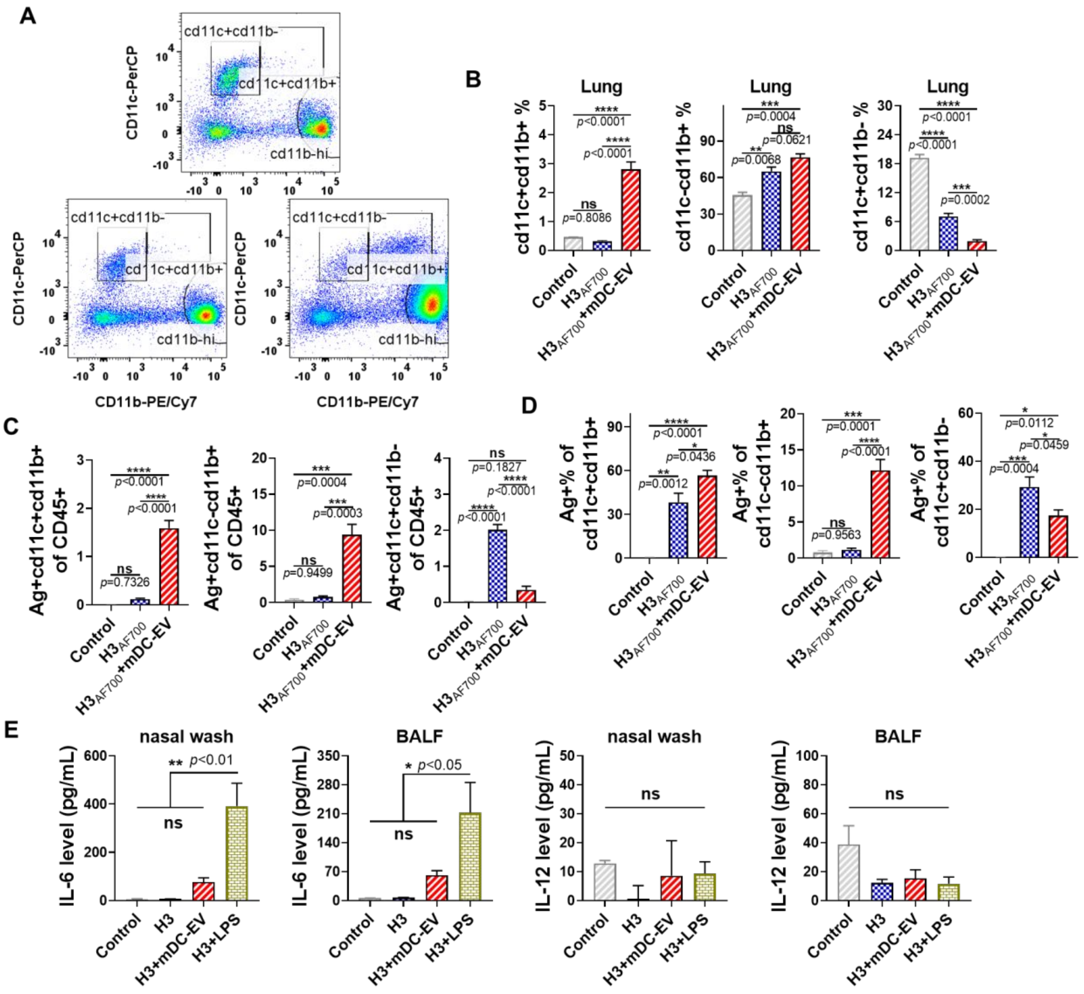

Fig. S10. Comparison of the non-lymphocyte immune cell subpopulations and lung antigen uptake behaviors one day post-immunization. (A-B) Comparison of the CD11c+CD11b-, CD11c+CD11b+, and CD11c-CD11b+ subpopulation frequencies between groups. (C) Frequencies of antigen-positive CD11c+CD11b-, CD11c+CD11b+, and CD11c-CD11b+ subpopulations among CD45+ non-lymphocytes. (D) Frequencies of antigen-positive cells among CD11c+CD11b-, CD11c+CD11b+, and CD11c-CD11b+ subpopulations. (E) The IL-6 and IL-12 levels in collected nasal washes and BALF. Data are presented as mean ± SEM (n = 3 for the control group and n = 4 for immunization groups). Statistical significance was analyzed by one-way ANOVA followed by Tukey's multiple comparison tests ($p > 0.05$, ns, not significant; $*p < 0.05$; $**p < 0.01$; $***p < 0.001$; $****p < 0.0001$).

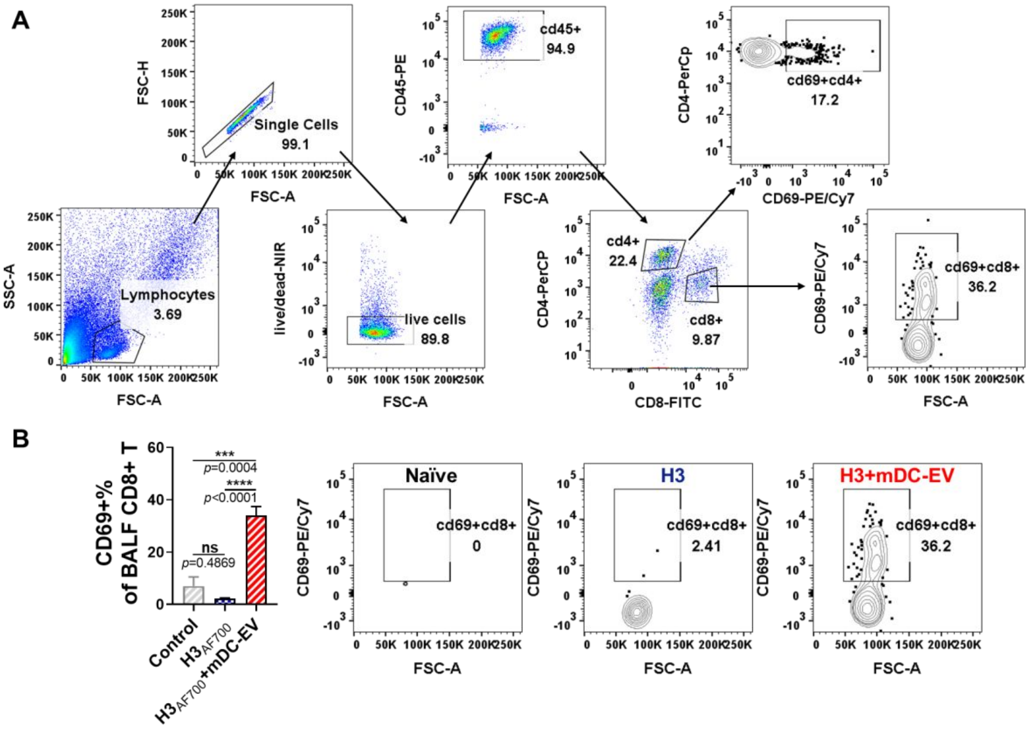

Fig. S11. T cell activation in BALF cells one day post-immunization. (A) Gating strategy. A lung sample from the H3+mDC-EV group was used as an example. (B) Frequencies of CD69+ cells among CD8+ T cells in BALF. Data are presented as mean ± SEM (n = 3 for the control group and n = 4 for immunization groups). Statistical significance was analyzed by one-way ANOVA followed by Tukey's multiple comparison tests ($p > 0.05$, ns, not significant; ***$p < 0.001$; ****$p < 0.0001$).

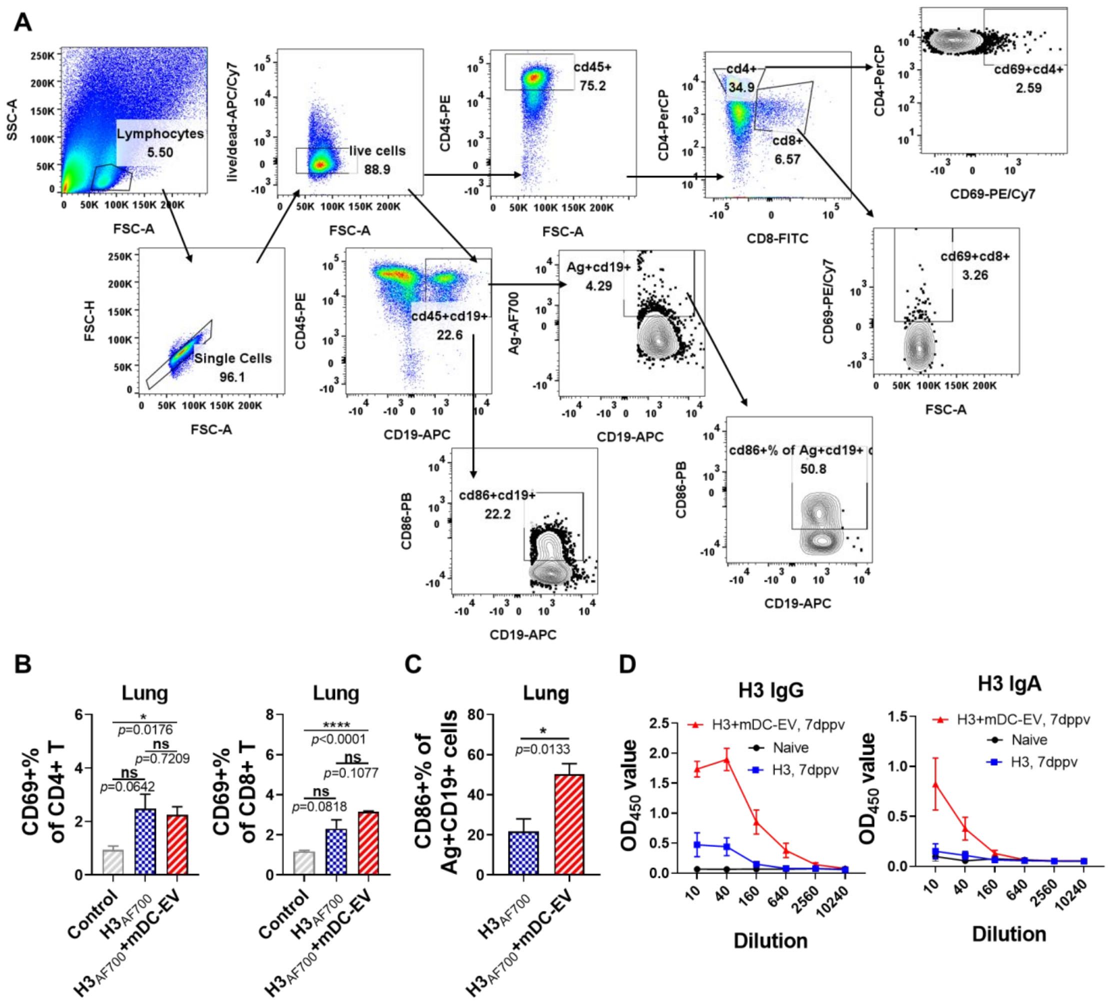

Fig. S12. T and B cell activation in lung cells one day post-immunization. (A) Gating strategy. A lung sample from an H3+mDC-EV-immunized mouse was used as a representative example. (B) Frequencies of CD69+ cells among CD4+ and CD8+ T cells in mouse lungs. (C) Frequencies of CD86+ cells among Ag+CD19+ lung B cells. Ag represents $H3_{AF700}$. (D) H3-specific IgG and IgA levels in immune sera 7 days post-priming immunization (7dppv). Data are presented as mean ± SEM (n = 3 for the control group and n = 4 for immunization groups). Statistical significance was analyzed by one-way ANOVA followed by Tukey's multiple comparison tests in B or Student's *t*-test in C (p > 0.05, ns, not significant; *p < 0.05; ****p < 0.0001).

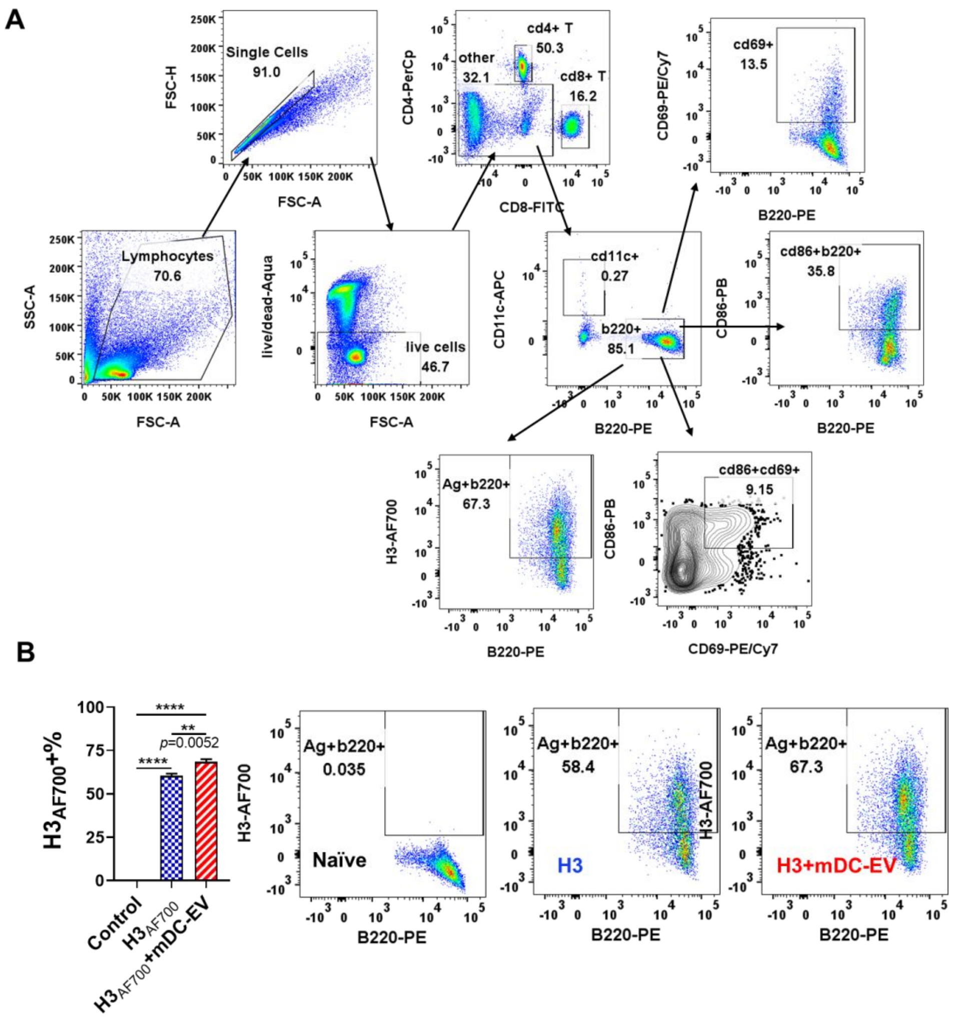

Fig. S13. Antigen uptake in splenic B cells and B cell activation. (A) Gating strategy. A sample from the H3+mDC-EV group was used as an example. (B) Quantification results of the $H3_{AF700}$-positive B220+ B cells. Data are presented as mean ± SEM (n = 3 for the control group and n = 4 for the immunization groups). Statistical significance was analyzed by one-way ANOVA followed by Tukey's multiple comparison tests (**p < 0.01; ****p < 0.0001).

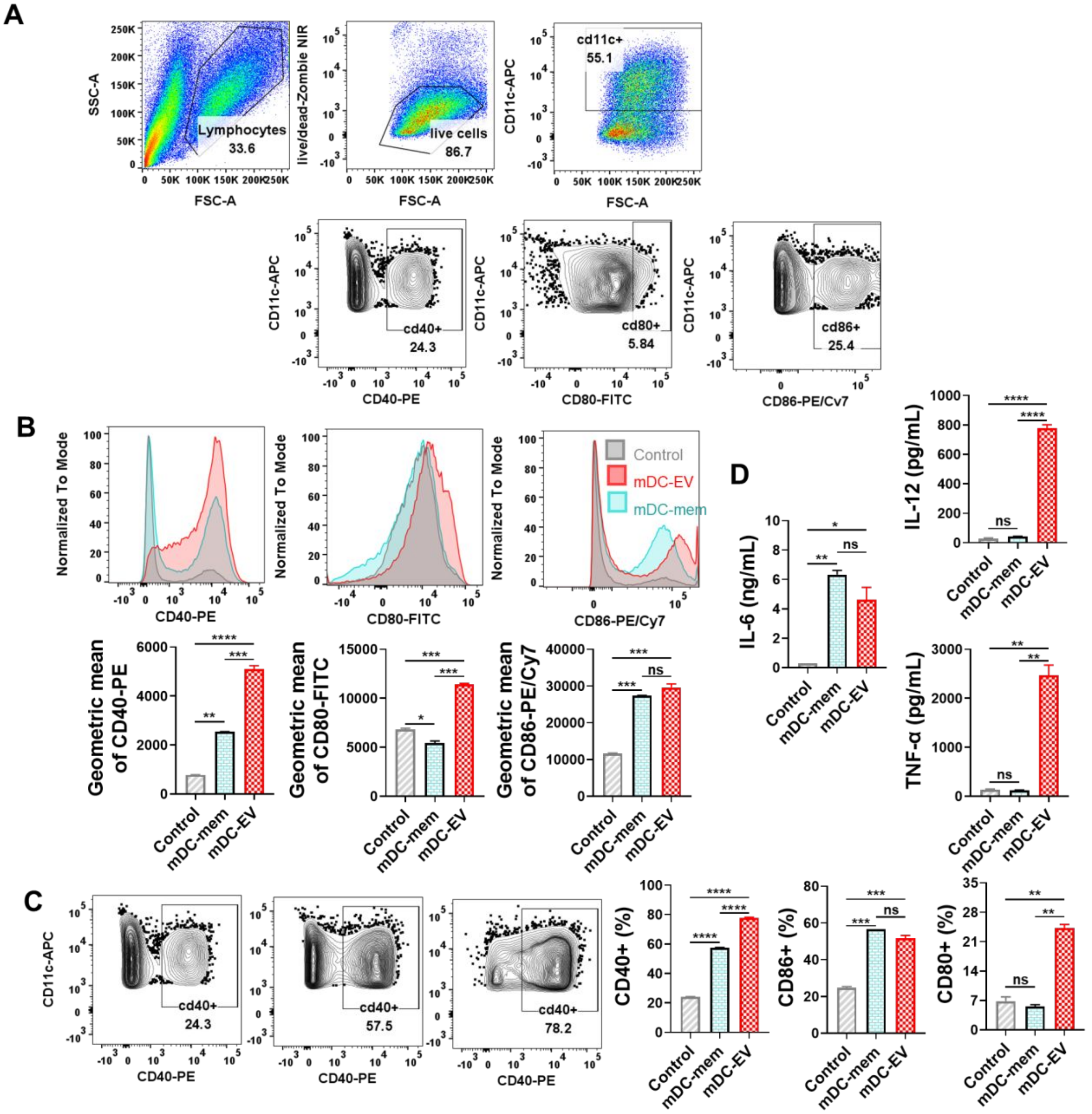

Fig. S14. Comparison of the immunostimulatory effects of mDC-EVs versus mDC-mem on BMDCs. (A) Gating strategy of BMDC cells. (B) Geometric mean fluorescence intensity of CD40, CD80, and CD86 on cultured BMDC cells by flow cytometry. (C) Frequencies of CD40-, CD80-, and CD86-positive cell populations. (D) Cytokine levels in cell culture supernatants. Data are presented as mean ± SEM. Duplicate samples were tested. Statistical significance was analyzed by one-way ANOVA followed by Tukey's multiple comparison tests ($p > 0.05$, ns, not significant; *$p < 0.05$; **$p < 0.01$; ***$p < 0.001$; ****$p < 0.0001$).

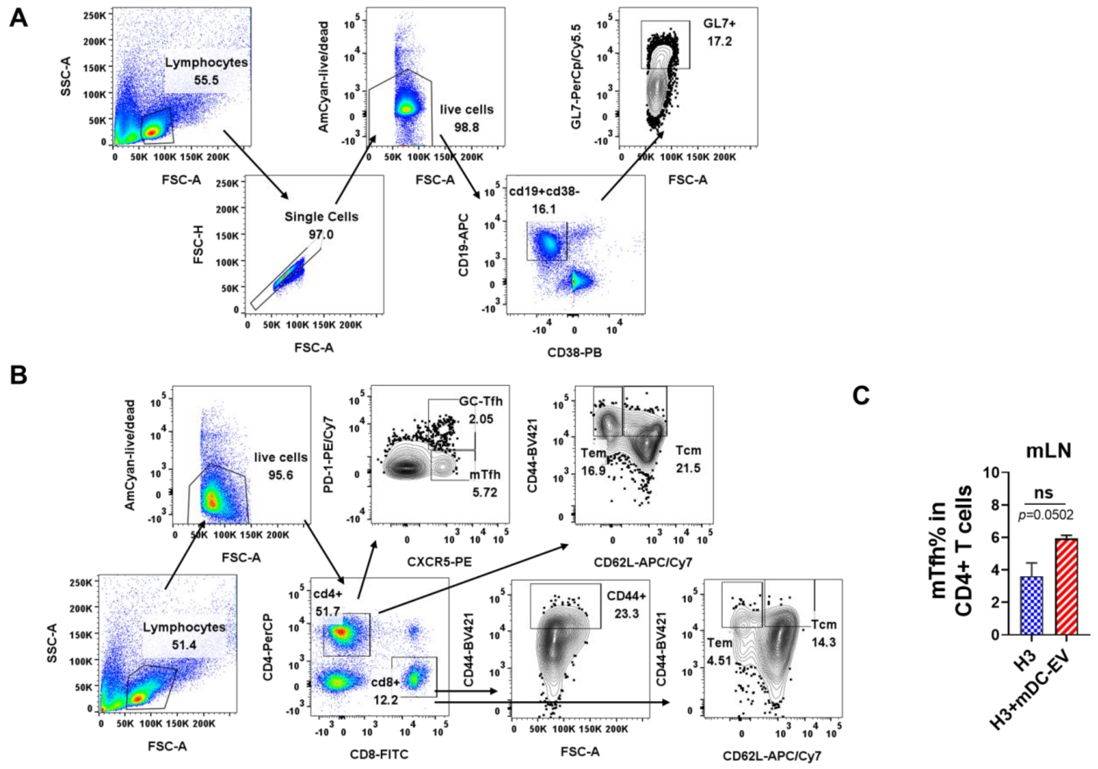

Fig. S15. Germinal center (GC) and T cell responses in mLNs 14 days post-immunization. (A) Gating strategy for CD19+CD38-GL7+ GC B cells. (B) Gating strategy for Tcm, Tem, and GC Tfh cells. (C) Frequencies of CD4+CXCR5+PD1- mTfh cells. Data are presented as mean ± SEM (n = 3). Statistical significance was analyzed by Student's t-test whenever applicable ($p > 0.05$, ns, not significant; *$p < 0.05$; **$p < 0.01$; ***$p < 0.001$; ****$p < 0.0001$).

Table S1. List of mouse antibodies for flow cytometry.

Antigen-Fluorescence	Clone	Catalog Number	Brand
CD45-PE	30-F11	103105	BioLegend
CD45-FITC	30-F11	103108	BioLegend
CD4-PerCP/Cyanine5.5	RM4-5	550954	BD Pharmingen™
CD8α-FITC	53-6.7	100712	BioLegend
CD44-BV421	IM7	103040	BioLegend
CD62L-APC/Cy7	MEL-14	104428	BioLegend
CD69-PE/Cy7	H1.2F3	104511	BioLegend
CD49a-PE	HMα1	142603	BioLegend
CD3e-PE	145-2c11	12-0031-82	eBioscience
CD127-APC	A7R34	135011	BioLegend
CXCR5-PE	L138D7	145503	BioLegend
CD279 (PD-1)-PE/Cy7		109109	BioLegend
CD19-APC	1D3	550992	BD Pharmingen™
CD45R/B220-AF700	RA3-6B2	103231	BioLegend
CD45R/B220-PE	RA3-6B2	103207	BioLegend
IgD-FITC	11-26c.2a	405703	BioLegend
CD38-Pacific Blue	90	102719	BioLegend
GL7-PerCP/Cy5.5	GL7	144609	BioLegend
CD11c-APC	HL3	550261	BD Pharmingen™
CD11c-PerCP	N418	117326	BioLegend
CD11b-PE/Cy7	M1/70	101216	BioLegend
Ly6C-BV510	HK1.4	128033	BioLegend
CD40-PE	3/23	124610	BioLegend
CD80-FITC	16-10A1	104706	BioLegend
CD86-APC/Cy7	GL-1	105030	BioLegend
CD86-PE/Cy7	GL-1	105013	BioLegend
CD86-PB	GL-1	105022	BioLegend
Zombie Aqua™ Fixable Viability Kit	n/a	423101	BioLegend
Zombie NIR Fixable Viability Kit	n/a	423105	BioLegend
CD16/32	2.4G2	553142	BD Pharmingen™
Note: FACS antibodies were diluted at 1:150 for cell staining.			

Table S2. List of mouse antibodies used in ELISA, ELISpot, and Western Blot assays

Antibody	Clone	Catalog/Lot Number	Brand
HRP-conjugated goat anti-mouse IgG	Polyclonal	Cat: 1033-05<br>Lot: J3316-P623D	SouthernBiotech
HRP-conjugated goat anti-mouse IgG1	Polyclonal	Cat: 1071-05, Lot: K1619-N733	SouthernBiotech
HRP-conjugated goat anti-mouse IgG2a	Polyclonal	Cat: 1080-05, Lot: B4520-RC63B	SouthernBiotech
HRP-conjugated goat anti-mouse IgA	Polyclonal	Cat:1040-05, Lot: J4416-M729	SouthernBiotech
anti-mouse IL-4	11B11	Cat: 504102	BioLegend
anti-mouse IL-2	JES6-1A12	Cat: 503704	BioLegend
anti-mouse IFN-γ	R4-6A2	Cat: 551216	BD Biosciences
anti-mouse IL-6	MP5-20F3	Cat: 504513	BioLegend
anti-mouse IL-12	C18.2	Cat: 511802	BioLegend
anti-mouse TNF-α	6B8	Cat: 510801	BioLegend
anti-mouse/rat IL-1β	B122	Cat: 503502	BioLegend
anti-human IL-1β	H1b-27	Cat: 511601	BioLegend
biotin-conjugated anti-mouse IL-4	BVD6-24G2	Cat: 504202	BioLegend
biotin-conjugated anti-mouse IL-2	JES6-5H4	Cat: 503804	BioLegend
biotin-conjugated anti-mouse IFN-γ	XMG1.2	Cat: 554410	BD Biosciences
biotin-conjugated anti-mouse IL-6	MP5-32C11	Cat: 504602	BioLegend
biotin-conjugated anti-mouse IL-12	C17.8	Cat: 505302	BioLegend
biotin-conjugated anti-mouse TNF-α	MP6-XT22	Cat: 506312	BioLegend
biotin-conjugated anti-mouse IL-1β	Poly5158	Cat: 515801	BioLegend
biotin-conjugated anti-human IL-1β	H1b-98	Cat: 511703	BioLegend
Note: HRP-conjugated goat anti-mouse IgG, IgG1, IgG2a, and IgA antibodies were used at a dilution of 1:3000 to 1:4000 for antibody ELISA assays, and at a dilution of 1:1000 for B-cell ELISpot assays. Biotin-conjugated cytokine detection antibodies were used at a dilution of 1:500 in T-cell ELISpot assays.			
